# Characterization of Arabidopsis CYP79C1 and CYP79C2 by Glucosinolate Pathway Engineering in *Nicotiana benthamiana* Shows Substrate Specificity Toward a Range of Aliphatic and Aromatic Amino Acids

**DOI:** 10.3389/fpls.2020.00057

**Published:** 2020-02-14

**Authors:** Cuiwei Wang, Mads Møller Dissing, Niels Agerbirk, Christoph Crocoll, Barbara Ann Halkier

**Affiliations:** ^1^DynaMo Center, Department of Plant and Environmental Sciences, Faculty of Science, University of Copenhagen, Frederiksberg, Denmark; ^2^Plant Biochemistry Section, Department of Plant and Environmental Sciences, University of Copenhagen, Frederiksberg, Denmark

**Keywords:** CYP79C1, CYP79C2, glucosinolates, oximes, cytochrome P450, metabolic engineering

## Abstract

Glucosinolates (GLSs) are amino acid-derived defense compounds characteristic of the Brassicales order. Cytochromes P450s of the CYP79 family are the entry point into the biosynthetic pathway of the GLS core structure and catalyze the conversion of amino acids to oximes. In *Arabidopsis thaliana*, CYP79A2, CYP79B2, CYP79B3, CYP79F1, and CYP79F2 have been functionally characterized and are responsible for the biosynthesis of phenylalanine-, tryptophan-, and methionine-derived GLSs, respectively. However, the substrate(s) for CYP79C1 and CYP79C2 were unknown. Here, we investigated the function of CYP79C1 and CYP79C2 by transiently co-expressing the genes together with three sets of remaining genes required for GLS biosynthesis in *Nicotiana benthamiana*. Co-expression of *CYP79C2* with either the aliphatic or aromatic core structure pathways resulted in the production of primarily leucine-derived 2-methylpropyl GLS and phenylalanine-derived benzyl GLS, along with minor amounts of GLSs from isoleucine, tryptophan, and tyrosine. Co-expression of *CYP79C1* displayed minor amounts of GLSs from valine, leucine, isoleucine, and phenylalanine with the aliphatic core structure pathway, and similar GLS profile (except the GLS from valine) with the aromatic core structure pathway. Additionally, we co-expressed *CYP79C1* and *CYP79C2* with the chain elongation and aliphatic core structure pathways. With the chain elongation pathway, CYP79C2 still mainly produced 2-methylpropyl GLS derived from leucine, accompanied by GLSs derived from isoleucine and from chain-elongated mono- and dihomoleucine, but not from phenylalanine. However, co-expression of *CYP79C1* only resulted in GLSs derived from chain-elongated amino acid substrates, dihomoleucine and dihomomethionine, when the chain elongation pathway was present. This shows that CYP79 activity depends on the specific pathways co-expressed and availability of amino acid precursors, and that description of GLS core structure pathways as “aliphatic” and “aromatic” pathways is not suitable, especially in an engineering context. This is the first characterization of members of the CYP79C family. Co-expression of CYP79 enzymes with engineered GLS pathways in *N. benthamiana* is a valuable tool for simultaneous testing of substrate specificity against multiple amino acids.

## Introduction

Glucosinolates (GLSs) are amino acid-derived specialized metabolites found in the Brassicales order including vegetables like cabbage, oil crops like canola and mustards as well as the model plant *Arabidopsis thaliana* (Halkier and Gershenzon, 2006). GLSs are hydrolyzed by myrosinase enzymes to form biologically active compounds such as isothiocyanates, nitriles, oxazolidine-2-thiones, and various indole derivatives. The hydrolysis products are deterrent or toxic to herbivores and pests, which makes GLSs play an important role in the native plant defense. From a human perspective, GLSs are flavor compounds, health-promoting agents, and biopesticides. A recent literature survey found 88 GLS structures that have been well characterized, 49 less well characterized and in some cases of uncertain existence, and many more candidates suggested and awaiting characterization (Blažević et al., 2020). In discussions of biosynthesis, it is meaningful to classify GLSs according to precursor amino acid. GLSs derived from five aliphatic standard amino acids (alanine, valine, leucine, isoleucine, and methionine) are well established. In addition, three aromatic amino acids serve as precursors for GLSs: tryptophan for indole GLSs, and phenylalanine and tyrosine for benzenic GLSs. All GLSs share a common core structure with a thioglucose moiety and a sulfated oxime. Diversity of GLSs is due to the variation of precursor amino acid, chain elongation of precursor amino acids, secondary modifications of amino acid side chain, and decoration on the glucose moiety. GLSs derived from chain-elongated amino acids are only known with certainty in the case of methionine, phenylalanine, and isoleucine (Blažević et al., 2020), but GLSs from once and twice chain elongated leucine have also been tentatively verified from nature.

The biosynthetic pathway of glucosinolates has three phases consisting of a side chain elongation, a core structure pathway and secondary modifications (Sønderby et al., 2010). For simplicity, the “core structure pathway” is shortened to the “core pathway” in the rest of this paper. The chain elongation pathway includes the enzymes BCAT4, MAM1-3, IPMI, and IPMDH1 (Sønderby et al., 2010). The core pathway is comprised of seven enzymatic steps. The substrate-specific cytochrome P450s of the CYP79 family constitute the entry point, catalyzing the conversion of amino acids to the corresponding oximes. Subsequently, downstream enzymes of the core pathway convert the oximes successively. While some enzymes in the core pathway are shared by all GLSs, other steps include several gene products with more limited substrate specificity or regulation. It is generally believed that some *A. thaliana* enzymes are mainly responsible for biosynthesis from aliphatic amino acids (mainly homologs of methionine), while others are mainly responsible for biosynthesis from tryptophan or possibly all aromatic amino acids (Sønderby et al., 2010). However, relatively little is still known of the exact enzymology of the individual core pathway enzymes. In *A. thaliana*, the downstream enzymes GGP1 and SUR1 are believed to be shared for all amino acid precursors. Generally, conversion of tryptophan or possibly all aromatic amino acids to GLSs is believed to additionally depend on CYP83B1, GSTF9, UGT74B1, and SOT16, and conversion of homo-methionine and possibly all aliphatic amino acids to GLSs is believed to depend on CYP83A1, GSTF11, UGT74C1, and SOT18 (Sønderby et al., 2010). For simplicity, we refer to these generally agreed groupings as the “aromatic” and “aliphatic” core pathways in the following, although our results suggest that these designations may be overly simplified.

Much is known about the enzymes that control the entry to GLS biosynthesis. In *A. thaliana*, CYP79A2 catalyzes conversion of phenylalanine to phenylacetaldoxime in the biosynthesis of benzyl GLS (BGLS), at least upon overexpression, while the role *in planta* remains poorly understood (Wittstock and Halkier, 2000). CYP79B2 and CYP79B3 catalyze conversion of tryptophan to indole-3-acetaldoxime for indole GLS biosynthesis (Mikkelsen et al., 2000; Zhao et al., 2002). CYP79F1 converts mono- to hexahomomethionine to the corresponding oximes for the biosynthesis of short- and long-chain methionine-derived GLSs whereas CYP79F2 exclusively accepts the long-chain homologs pentahomo- and hexahomomethionine (Chen et al., 2003). However, the function of two additional *A. thaliana* enzymes, CYP79C1 and CYP79C2, remained unknown, although based on sequence similarity to other CYP79s they were anticipated to control entry to GLS biosynthesis.

Expression levels of *CYP79C1* and *CYP79C2* have been investigated by several authors using transcriptomics analysis in *A. thaliana*. Under normal growth conditions, 14 days after germination, *CYP79C1* and *CYP79C2* were not expressed (Capovilla et al., 2018; Nielsen et al., 2019). Generally, the expression levels of *CYP79C1* and *CYP79C2* are below the detection limit level in vegetative parts (root, leaf, stem) at all developmental stages (Klepikova et al., 2016). However, *CYP79C1* is expressed in floral organs (e.g. ovules, flowers, and seeds) (Col-0 accession) and *CYP79C2* is expressed in embryo central cells (Landsberg *erecta* accession) (Schmid et al., 2012; Klepikova et al., 2016).

In this study, we identified catalytic functions of CYP79C1 and CYP79C2 from *A. thaliana* by *Agrobacterium*-mediated transient expression in *Nicotiana benthamiana*. Each CYP79 enzyme was expressed together with the downstream enzymes of the aromatic and aliphatic core pathways as well as the enzymes of the chain elongation pathway, followed by analysis of GLS profiles. The CYP79Cs primarily resulted in GLSs derived from leucine and phenylalanine when the chain elongation pathway was absent, but excluded phenylalanine and included homologs of methionine and leucine when the chain elongation pathway was present. Differential effects of the “aliphatic” and “aromatic” core pathways were observed. Engineering the GLS biosynthetic pathways in *N. benthamiana* is a novel, untargeted approach to characterize CYP79 enzymes.

## Materials and Methods

### Generation and Transformation of Constructs

All constructs were cloned from the vector pCAMBIA330035Su by USER cloning (Nour-Eldin et al., 2006; Geu-Flores et al., 2007). The NEB^®^ DH10B strain (New England Biolabs, #C3019H) was used to assemble and amplify the constructs. The gene sequences of CYP79D2 (NC_035172.1), CYP79F1 (AT1G16410), CYP83A1 (AT4G13770), CYP83B1 (AT4G31500), and GGP1 (AT4G30530) were amplified with in-house templates. The gene sequences of CYP79C1 (AT1G79370) and CYP79C2 (AT1G58260) were amplified from gBlocks from IDT (Integrated DNA Technologies Inc, USA). All genes were inserted into the vector pCAMBIA330035Su flanked with 35S promoter and 35S terminator. Constructs C11 containing the genes *GSTF11* and *GGP1*, and C10 containing the genes *SUR1*, *UGT74C1*, and *SOT18* were previously published (Mikkelsen et al., 2010). The construct with *APK2* gene was published by Møldrup et al. (2011). The construct ORF2 harboring the genes *SUR1*, *UGT74B1*, and *SOT16* and the construct ORF1-GGP1 harboring the genes *CYP79A2*, *CYP83B1*, and *GGP1* were described in (Geu-Flores et al., 2009). The chain elongation pathway constructs BCAT4 (AT3G19710), BAT5 (AT4G12030), MAM1 (AT5G23010), IPMI-LSU1 (AT4G13430), IPMI-SSU3 (AT3G58990), and IPMDH1 (AT5G14200) were from the previous study (Crocoll et al., 2016b). The primers used in this study are summarized in **Supplementary Table S1**.

All constructs were separately transformed into *Agrobacterium tumefaciens* strain pGV3850 by electroporation (2 mm cuvette, 2.5 kV, 400 Ω, and 25 μF) in a Bio-Rad GenePulser (Bio-Rad, Hercules, CA, USA). Cells were incubated at 28°C for 2 h after 200 µl LB media was added. The cultures were plated on LB agar plates containing 30 μg/ml rifampicin and 50 μg/ml kanamycin and incubated at 28°C for 3 days. Colony PCR was used to confirm the presence of the constructs in the strain.

### Transient Expression in *N. benthamiana*

*Agrobacterium tumefaciens* strains harboring the different constructs were grown in YEP media containing 30 μg/ml rifampicin and 50 μg/ml kanamycin at 28°C and 220 rpm overnight. Cells were harvested by centrifugation at 4000 × *g* for 10 min at room temperature. Subsequently, the pellets were resuspended in infiltration buffer (10 mM MES, 10 mM MgCl_2_, pH 5.6) with 100 μM acetosyringone (3,5-dimethoxy-4-hydroxyacetophenone, Sigma-Aldrich, Steinheim, Germany) and slightly shaken at room temperature for 1–3 h. OD_600_ for each culture was measured and infiltration buffer was added to adjust to OD_600_ = 0.2. Equal volumes of each infiltration buffer containing individual construct were mixed according to the combination design. For expression of the core pathway, the proper amount of infiltration buffer was added for the combinations with fewer than six constructs, resulting in each individual construct with OD_600_ ≈ 0.03. For expression of the chain elongation pathway and the core pathway, the proper amount of infiltration buffer was added for the combinations with fewer than 12 constructs, resulting in each individual construct with OD_600_ ≈ 0.017. The silencing suppressor p19 (Voinnet et al., 2003) was included in all experiments. Leaves of *N. benthamiana* plants (around 4 weeks old, four to six leaves stage) were infiltrated using the mixed cultures of the different combinations.

### Plant Extraction and GLS Analysis

Four leaf disks of 1 cm diameter were harvested from each infiltrated leaf and weighed 5 days after infiltration. Metabolites were extracted from the leaf disks using 85% aq. methanol. The resulting extract was diluted 5.0 fold with water and the diluted samples were directly analyzed by LC-MS/qTOF for identification of native GLSs. For quantification, GLSs were analyzed as desulfo-GLSs (dsGLSs) after enzymatic on-column desulfation as previously described (Jensen et al., 2015; Crocoll et al., 2016a). Allyl GLS (K^+^ salt, PhytoLab, Vestenbergsgreuth, Germany) was added as internal standard before desulfation with a final concentration of 1 µM. Subsequently, the extract was loaded onto DEAE-Sephadex columns to bind GLSs. Columns were washed twice with 70% methanol and twice with water. After sulfatase treatment overnight, dsGLSs were eluted with water.

### GLS Analysis by Desulfation and LC-MS/Triple Quadrupole

GLSs were analyzed as dsGLSs after enzymatic desulfation as previously described (Jensen et al., 2015; Crocoll et al., 2016a) with modifications for separation of leucine and isoleucine-derived dsGLSs. Briefly, chromatography was performed on an Advance UHPLC system (Bruker, Bremen, Germany). Separation was achieved on a Kinetex 1.7u XB-C18 column (100 x 2.1 mm, 1.7 µm, 100 Å, Phenomenex, Torrance, CA, USA). Formic acid (0.05%) in water and acetonitrile (supplied with 0.05% formic acid) were employed as mobile phases A and B, respectively. An extended elution profile was used: 0–0.5 min, 2% B; 0.5–3.2 min, 2–30% B; 3.2–4.0 min 30–100% B, 4.0–4.5 min 100% B, 4.5–4.6 min, 100–2% B, and 4.6–6.0 min 2% B. The mobile phase flow rate was 400 µl min^−1^. The column temperature was maintained at 40°C. The liquid chromatography was coupled to an EVOQ Elite TripleQuad mass spectrometer (Bruker, Bremen, Germany) equipped with an electrospray ion source (ESI) operated in positive ionization mode. The instrument parameters were optimized by infusion experiments with pure standards. The ion spray voltage was maintained at +3500 V. Cone temperature was set to 350°C and cone gas to 20 psi. Heated probe temperature was set to 400°C and probe gas flow to 40 psi. Nebulizing gas was set to 60 psi and collision gas to 1.6 mTorr. Nitrogen was used as probe and nebulizing gas and argon as collision gas. Active exhaust was constantly on. Multiple reaction monitoring (MRM) was used to monitor analyte precursor ion to product ion transitions: MRM transitions for phenylalanine-, tryptophan, and tyrosine-derived ds-GLSs and ds-GLS derived from chain-elongated methionine were chosen as previously reported (Crocoll et al., 2016a; Petersen et al., 2019a) and from LC-MS/qTOF data for valine-, leucine-, and isoleucine-derived ds-GLSs as well as ds-GLS derived from chain-elongated leucine. Details on transitions and collision energies are described in **Supplementary Table S2**. Both Q1 and Q3 quadrupoles were maintained at unit resolution. Bruker MS Workstation software (Version 8.2.1, Bruker, Bremen, Germany) was used for data acquisition and processing. Allyl GLS was used as internal standard for quantification as previously described (Jensen et al., 2015). Authentic references of ds1ME, ds1MP, and ds2MP were obtained as previously reported (Olsen et al., 2016). The response factors representing the relationship between the internal standard allyl GLS and 1ME, 1MP, and 2MP were set to 1 as amounts of the pure references were insufficient for accurate determination of mass by weighing.

### Confirmation of GLS Identity by LC-MS of Intact as Well as Desulfo-GLSs

The quantification of GLSs after desulfation is specific for each isomeric side chain structure, but does not provide a critical test of the correct position of the sulfate group in the native metabolites. Hence, intact GLS analysis was additionally performed. In general, where authentic references were available, correct positioning of the sulfate group as well as the general metabolite identity was confirmed by LC-MS/Q-TOF analysis (**Supplementary Methods** and **Supplementary Figures S2–S8**). However, in one positive control with very high levels of 1ME, peak broadening suggested additional accumulation of an isomer. Minor levels of this isomer were also observed in experiments with CYP79C1. Since peak broadening was not observed for authentic intact 1ME or desulfated GLSs (**Supplementary Figure S8**), we suggest that the peak broadening was due to heterogeneity of sulfation, possibly due to an endogenous sulfotransferase in tobacco. However, both isomers obviously reflected biosynthesis of the ds-GLS core structure, so the heterogeneity was not a problem for the characterization of the CYP79C enzymes. We isolated authentic 1ME as described (Agerbirk et al., 2014) (**Supplementary Figure S1**). For 1MP and 2MP, for which authentic references of intact GLSs were not available, the retention times (**Supplementary Figure S2** and **S5**) and fragmentations (results not shown) were as expected for GLSs, suggesting that these were likewise correctly sulfated. Identities of all reported GLSs were subsequently confirmed by comparison of retention time, accurate mass, and fragmentation patterns from MS2 experiments, including distinction of the isomers 1MP and 2MP with different retention times as dsGLSs and GLSs (**Supplementary Table S2**).

## Results

### GLS Production by Co-Expression of *CYP79C1* and *CYP79C2* With the Aliphatic Core Pathway

**Figure 1:**
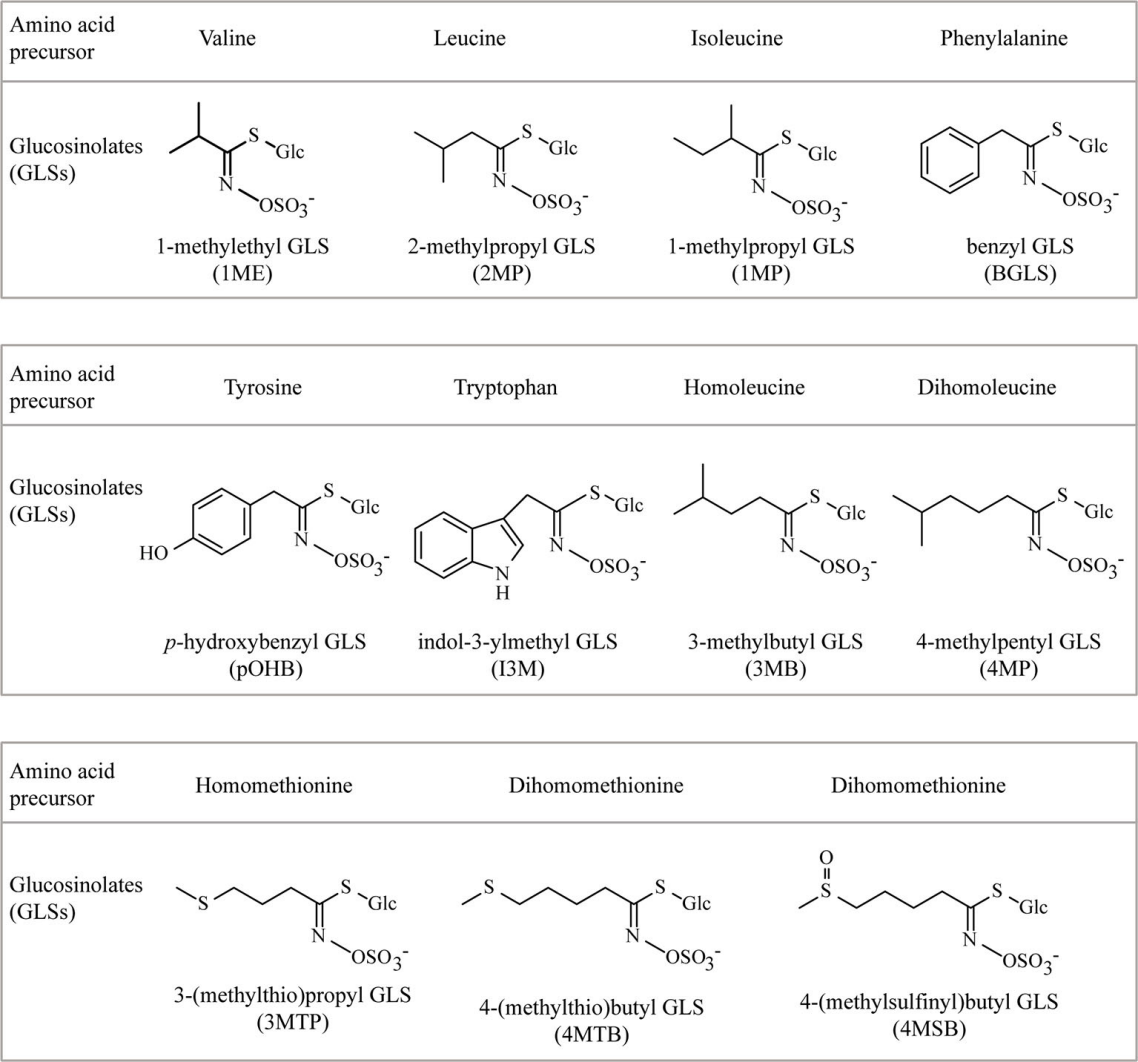
The chemical structures and amino acid precursors of all the glucosinolates (GLSs) detected in this study. Side-chain structures of 3MB and 4MP were deduced from *m*/*z* value and a biochemical argument (known chain elongation of Leu, not Ile, by the chain elongation enzymes used).

**Figure 2:**
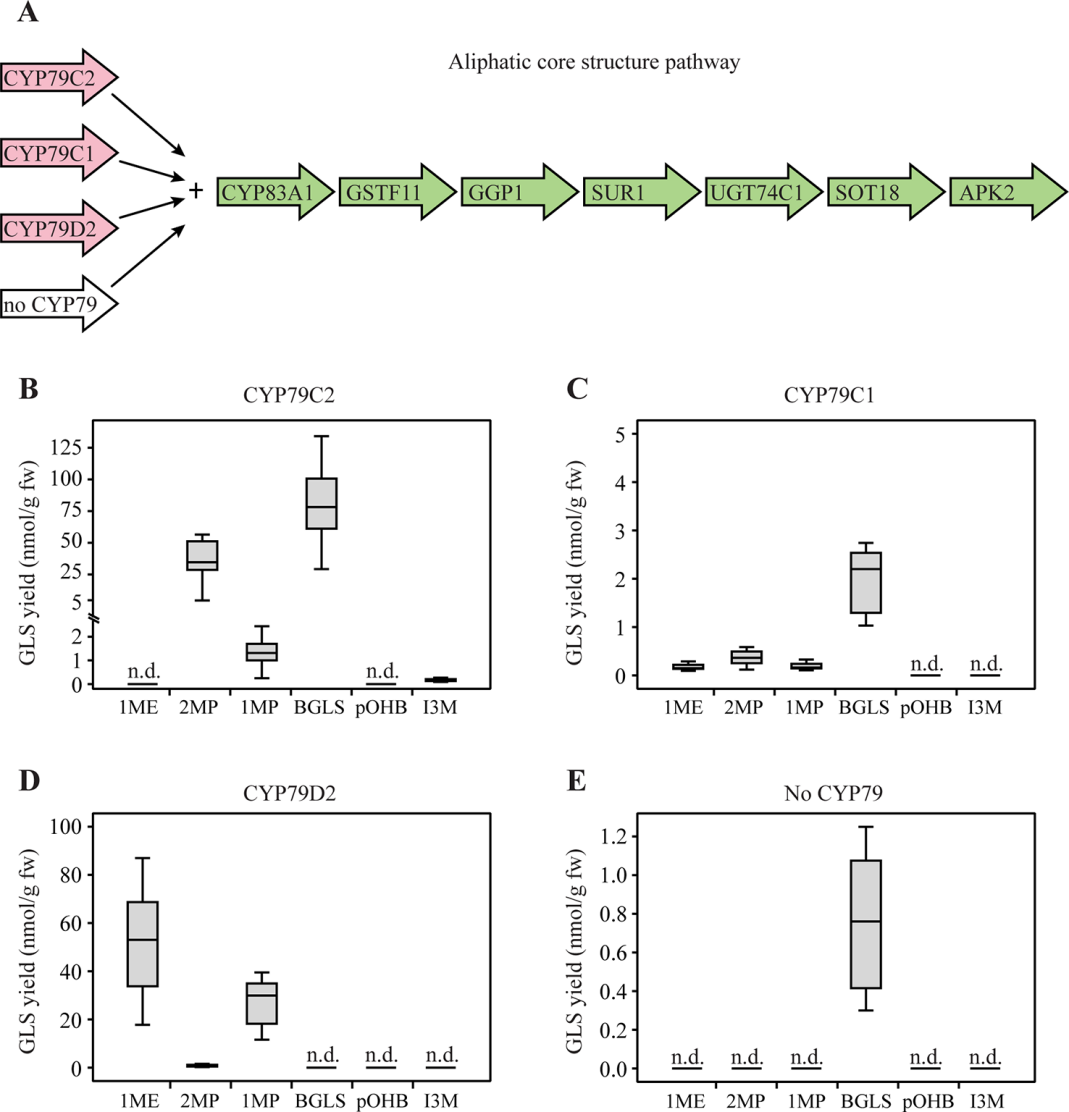
Glucosinolates (GLSs) accumulated in *Nicotiana benthamiana* upon transient expression of *CYP79C1* and *CYP79C2* in combination with the aliphatic core pathway. **(A)** Scheme of introduced enzymes including different CYP79 enzymes (pink) and remaining “aliphatic” core pathway enzymes as well as the stimulating enzyme APK2 (green). The combination without CYP79 represents the negative control and the combination with CYP79D2 is the positive control. **(B)** GLSs accumulated with construct including CYP79C2. **(C)** GLSs accumulated with constructs including CYP79C1. **(D)** GLSs accumulated with constructs including CYP79D2. **(E)** GLSs accumulated with the negative control constructs without CYP79 co-expressed. Data of each box plot represent nine biological replicates in nanomole per gram fresh weight. n.d. represents not detected. Data in **(B–E)** are box-and-whisker representations indicating the 10th (lower whisker), 25th (base of box), 75th (top of box), and 90th (top whisker) percentilies. The line within the box is the median and outliers are not shown. Exact values are listed in **Supplementary Table S3**. *P-*values (two-sided Student's *t*-test) used to compare the same type of GLS yields among B, C, and E are listed in **Supplementary Table S4**.

To investigate which GLSs are produced by CYP79C1 and CYP79C2, and thereby the substrate specificity of the two enzymes, we first co-expressed *CYP79C1* and *CYP79C2* with the remaining genes of the aliphatic core pathway in *N. benthamiana* (**Figure 2A**). In addition, the APS kinase gene *APK2* from *Arabidopsis* was co-expressed alongside the core pathway genes as it has been shown to be critical for efficient regeneration of the co-factor PAPS (3'-phospho-adenosine-5'-phosphosulfate) in the final sulfotransferase step that converts dsGLS into intact GLS (Møldrup et al., 2011). The chemical structures and amino acid precursors of all the GLSs detected in this study are shown in **Figure 1**. Co-expression with *CYP79C2* resulted in high accumulation of leucine-derived 2MP (35 nmol/g fw) and phenylalanine-derived BGLS (76 nmol/g fw) as well as low levels of isoleucine-derived 1MP (1.2 nmol/g fw) and tryptophan-derived I3M (0.16 nmol/g fw) (**Figure 2B** and **Supplementary Table S3**). When *CYP79C1* was co-expressed, accumulation of 2MP, 1MP and BGLS was observed (**Figure 2C**). The overall levels of the three GLSs were much lower, with the highest level being BGLS at 2.0 nmol/g fw (**Supplementary Table S3**). Additionally, a tiny amount of valine-derived 1ME was observed with *CYP79C1* (**Figure 2C**). Co-expression of *CYP79D2* (a cyanogenic CYP from *Manihot esculenta*) together with the aliphatic core pathway, was included as positive control and resulted in valine-derived 1ME and isoleucine-derived 1MP (**Figure 2D**), which is consistent with a previous report (Mikkelsen and Halkier, 2003). Additionally, leucine-derived 2MP was detected at very low levels (**Figure 2D**), suggesting that CYP79D2 has a low degree of acceptance of leucine as a substrate. A small but statistically significant amount of BGLS (0.76 nmol/g) was detected from the core pathway without any CYP79 enzyme added (**Figure 2E** and **Supplementary Table S3**), suggesting that an endogenous enzyme, possibly of the CYP79 family, from *N. benthamiana* is able to produce the phenylacetaldoxime that is further converted by the core pathway. Interestingly, this background level of BGLS was not observed with CYP79D2, maybe due to some degree of substrate competition. Hence, background levels from control experiments were not subtracted from reported levels from experiments with inserted CYP79 genes. The high level of the aromatic BGLS that accumulated upon co-expression of CYP79C2 with the “aliphatic” core pathway was unexpected, suggesting that the enzymes in the core pathway are less side chain specific than hitherto believed.

### GLS Production by Co-Expression of *CYP79C1* and *CYP79C2* With the Aromatic Core Pathway

**Figure 3:**
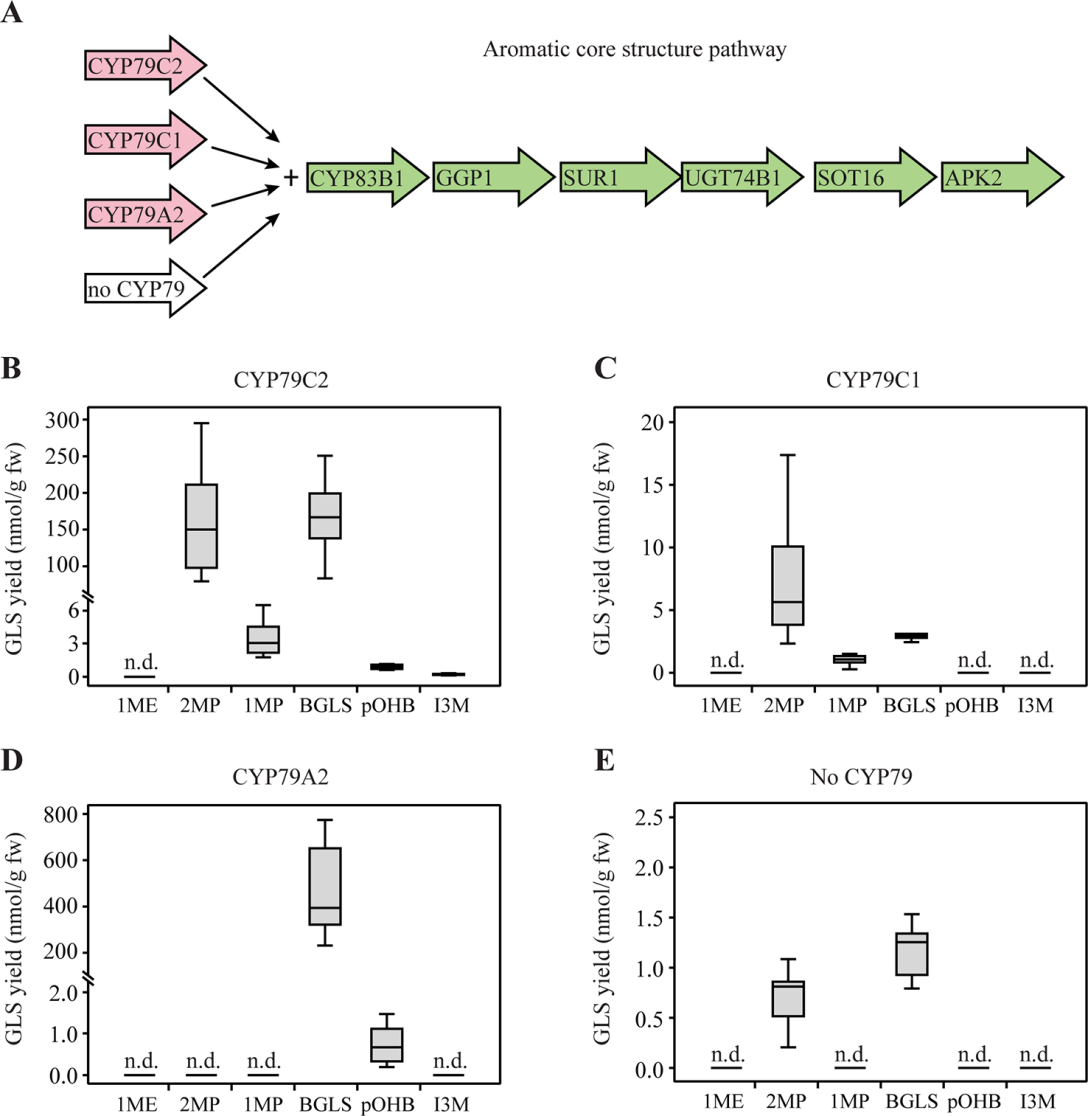
Glucosinolates (GLSs) accumulated in *Nicotiana benthamiana* upon transient expression of *CYP79C1* and *CYP79C2* in combination with the aromatic core pathway. **(A)** Scheme of introduced enzymes including different CYP79 enzymes (pink) and remaining “aromatic” core pathway enzymes as well as the stimulating enzyme APK2 (green). The combination without CYP79 represents the negative control and the combination with CYP79A2 is the positive control. **(B)** GLSs accumulated with constructs including CYP79C2. **(C)** GLSs accumulated with constructs including CYP79C1. **(D)** GLSs accumulated with constructs including CYP79A2. **(E)** GLSs accumulated with the negative control constructs without CYP79 co-expressed. Data of each box plot represent nine biological replicates in nanomole per gram fresh weight. n.d. represents not detected. Data in **(B–E)** are box-and-whisker representations indicating the 10th (lower whisker), 25th (base of box), 75th (top of box), and 90th (top whisker) percentiles. The line within the box is the median and outliers are not shown. Exact values are listed in **Supplementary Table S5**. *P-*values (two-sided Student's *t*-test) used to compare the same type of GLS yields among B, C, and E are listed in **Supplementary Table S6**.

Next, we co-expressed *CYP79C1* and *CYP79C2* together with the genes of the aromatic core pathway in *N. benthamiana* (**Figure 3A**). The gene *GSTF9* was not co-expressed in this experiment since an endogenous activity in *N. benthamiana* was known to efficiently catalyze this step (Geu-Flores et al., 2009). In the experiments with *CYP79C2*, the observed GLS profile was very similar to the one observed from co-expression with the aliphatic core pathway, except that levels were generally higher and that minute amount of the tyrosine-derived pOHB was also detected (**Figure 3B**). More 2MP (180 nmol/g fw) and BGLS (169 nmol/g fw) were produced than 1MP (3.5 nmol/g fw), pOHB (0.97 nmol/g fw), and I3M (0.24 nmol/g fw) from co-expression of *CYP79C2* (**Supplementary Table S5**). Noticeably, the level of the aliphatic 2MP derived from leucine was much higher using the aromatic core pathway than the aliphatic core pathway (**Supplementary Tables S3** and **S5**). This supports the finding above that the enzymes in core pathways are less side chain specific than hitherto believed. Co-expression of *CYP79C1* produced a GLS profile similar to the profile obtained with the aliphatic core pathway, i.e. 2MP, 1MP, and BGLS, with the exception that 1ME was not produced (**Figure 3C**). The positive control for engineering the aromatic core pathway, CYP79A2, produced BGLS (480 nmol/g fw) and tiny amounts of pOHB (0.77 nmol/g fw) (**Supplementary Table S5** and **Figure 3D**). This result was in agreement with the previous conclusion that phenylalanine is the main substrate of CYP79A2 (Wittstock and Halkier, 2000), but extended the known substrate profile to include tyrosine when presented by a physiological mix of the various amino acids. The lack of observed pOHB by previous authors is apparently due to the much increased sensitivity of the analytical instrumentation used here. Finally, small amounts of 2MP and BGLS were observed from the negative control, i.e. the core pathway without CYP79 enzymes (**Figure 3E**). This result suggests that an endogenous enzyme, possibly of the CYP79 family, can produce the corresponding oximes, to be converted by the remaining enzymes of the aromatic core pathway to 2MP and BGLS. The background level of 2MP was not observed in the positive control with co-expression of CYP79A2, which is in agreement with a similar observation in the experiments with the aliphatic core pathway.

### CYP79C1 and CYP79C2 Also Metabolize Chain-Elongated Amino Acids

**Figure 4:**
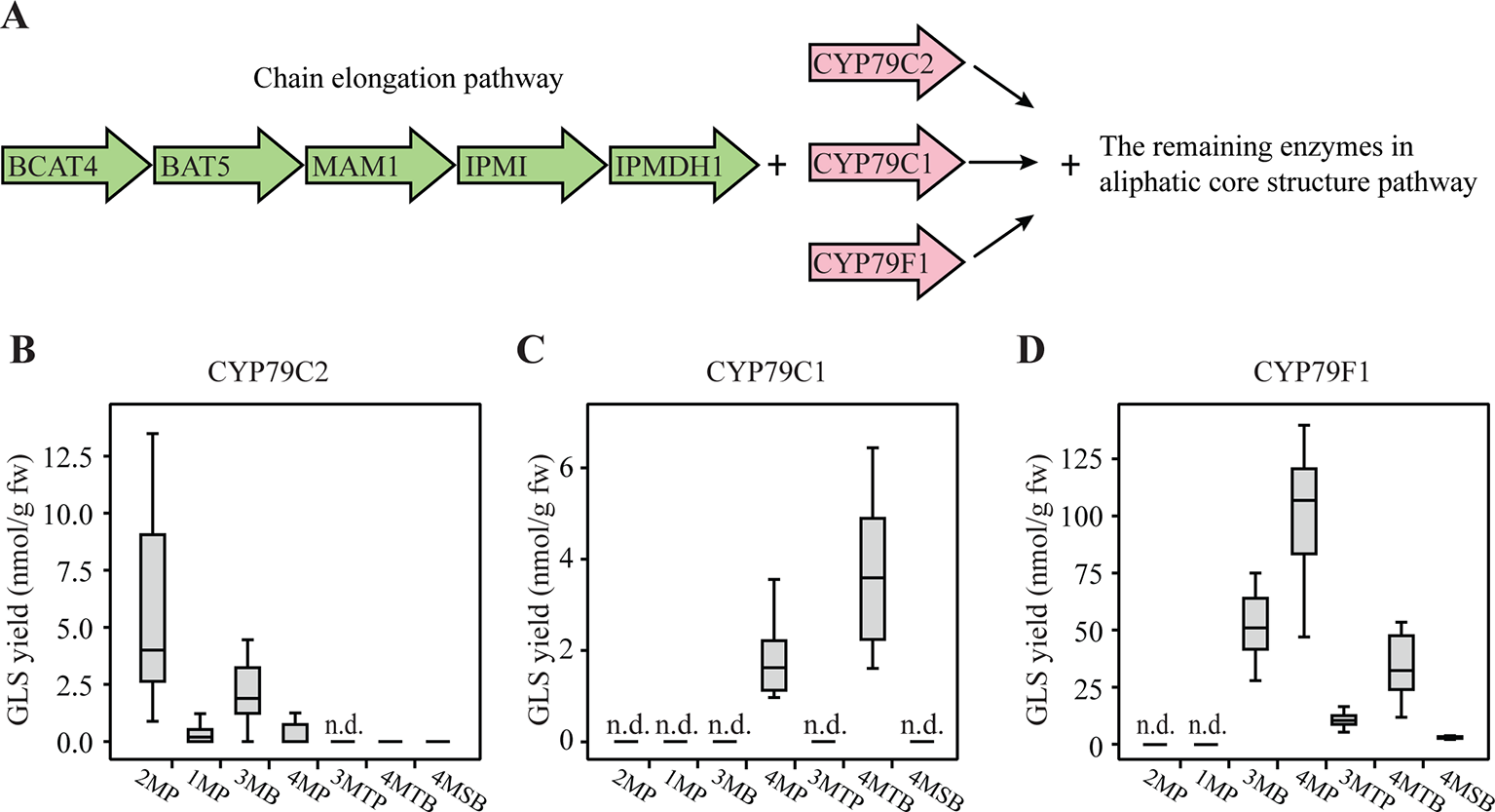
Glucosinolates (GLSs) accumulated in *Nicotiana benthamiana* upon transient expression of *CYP79C1* and *CYP79C2* in combination with the chain elongation and aliphatic core pathways. **(A)** Scheme of introduced enzymes including the chain elongation pathway enzymes (green), different CYP79 enzymes (pink), and the remaining “aliphatic” core pathway enzymes as well as the stimulating enzyme APK2. The combination with CYP79F1 is the positive control. **(B)** GLSs accumulated with constructs including CYP79C2. **(C)** GLSs accumulated with constructs including CYP79C1. **(D)** GLSs accumulated with constructs including CYP79F1. Data of each box plot represent 12 biological replicates in nanomole per gram fresh weight. n.d. represents not detected. The aromatic GLSs BGLS, pOHB, and I3M were not detected in any of these experiments, and are hence not indicated in the graphs. Data in **(B–D)** are box-and-whisker representations indicating the 10th (lower whisker), 25th (base of box), 75th (top of box), and 90th (top whisker) percentiles. The line within the box is the median and outliers are not shown. Exact values are listed in **Supplementary Table S7**. *P-*values (two-sided Student's *t*-test) used to compare the same type of GLS yields between B and C are listed in **Supplementary Table S8**.

To investigate whether also chain-elongated amino acids are metabolized by CYP79C1 and CYP79C2, we co-expressed *CYP79C1* and *CYP79C2* with the remaining enzymes of the aliphatic core pathway and the chain elongation pathway in *N. benthamiana* (**Figure 4A**). The chain elongation pathway has been reported to elongate the side chain of methionine in 1–3 cycles, leucine in 1–2 cycles and phenylalanine in one cycle, resulting in production of homomethionine, dihomomethionine, trihomomethionine, homoleucine, dihomoleucine, and homophenylalanine in *Escherichia coli* (Petersen et al., 2019b). As chain elongation of isoleucine was not detected with this specific set of chain elongation genes, we deduced that produced alkyl GLS could only be the isomers expected from leucine, i.e. with one branching methyl group at the “ω minus 1”-position (either homoleucine-derived 3-methylbutyl GLS, 3MB, or dihomoleucine-derived 4-methylpentyl GLS, 4MP) (**Figure 1**). Furthermore, we included co-expression of the bile acid transporter 5 gene (*BAT5*), as BAT5 facilitates transport of chain-elongated compounds between chloroplast and the cytosol (Crocoll et al., 2016b). We found that co-expression of *CYP79C2* at these conditions resulted in low levels of 3MB at 2.1 nmol/g fw and 4MP at 0.45 nmol/g fw (**Figure 4B** and **Supplementary Table S7**), indicating that CYP79C2 accepts homoleucine and dihomoleucine as substrates. Moreover, trace levels of dihomomethionine-derived 4-(methylthio)butyl GLS (4MTB) and 4-(methylsulfinyl)butyl GLS (4MSB) were detected (**Figure 4B**), but only in a few biological replicates (**Supplementary Table S7**), suggesting that CYP79C2 may occasionally accept dihomomethionine as substrate. Interestingly, 2MP and 1MP derived from non-chain-elongated amino acids accumulated, but the levels were much lower than those detected from CYP79C2 without the chain elongation pathway (**Supplementary Tables S3**, **S5** and **S7**). On the contrary, BGLS (also from non-chain-elongated phenylalanine) was not detected at all in these experiments. These results suggest that the *in vivo* activity of CYP79C2 depends on the presence or absence of the chain-elongation machinery. Co-expression of *CYP79C1* resulted in 4MP at 1.8 nmol/g fw and 4MTB at 3.7 nmol/g fw, suggesting that the substrate specificity of CYP79C1 includes dihomoleucine and dihomomethionine (**Figure 4C** and **Supplementary Table S7**). For CYP79C1, the effect of co-expression with the chain elongation pathway was even more pronounced, as no GLSs derived from non-chain-elongated amino acids were detected (**Figure 4C**). The positive control to validate the function of the entire pathway, CYP79F1, showed high accumulation of 51 nmol/g fw 3MB, 100 nmol/g fw 4MP, and 33 nmol/g fw 4MTB, as well as low level of 9.6 nmol/g fw 3-(methylthio)propyl GLS (3MTP) and 1.7 nmol/g fw 4MSB (**Figure 4D** and **Supplementary Table S7**). Trace amounts of trihomomethionine-derived 5-(methylsulfinyl)-pentyl GLS (5MSP) were detected (data not shown). This demonstrates that CYP79F1 catalyzed the conversion of homoleucine, dihomoleucine, homomethionine, dihomomethionine, and trihomomethionine to the corresponding oximes, as previously shown (Mikkelsen et al., 2010; Petersen et al., 2019b).

## Discussion

In this study, we characterized the substrate specificity of CYP79C1 and CYP79C2 by transiently co-expressing the genes together with the GLS biosynthetic pathways in *N. benthamiana*. From subsequent analysis of GLS profile, we deduced the substrate use of the tested CYP79s. Unexpectedly, it turned out that the resulting GLS profile depended strongly on the co-expressed pathway, either the “aromatic” or the “aliphatic” core pathway with or without the chain elongation pathway in addition.

In the absence of the chain elongation pathway, we found that CYP79C1 and CYP79C2 both channeled aliphatic leucine and isoleucine and aromatic phenylalanine into the corresponding GLS. For CYP79C2, leucine and phenylalanine were major and isoleucine was a minor substrate, independent of core pathway. For CYP79C1, the preferred substrate depended on the core pathway: aromatic phenylalanine was the sole major substrate with the “aliphatic” core pathway, while aliphatic leucine was the sole major substrate with the “aromatic” core pathway. In most cases, various minor substrates were also used. However, when the aliphatic core pathway was supplemented with the chain elongation pathway, neither of the two CYP79Cs used phenylalanine as precursor at all. Other differences were also observed: CYP79C2 showed substrate specificity to homo- and dihomoleucine, while CYP79C1 stopped metabolizing non-chain-elongated amino acids and apparently exclusively channeled dihomoleucine and dihomomethionine into GLS biosynthesis (**Figures 2**–**4**, Bassard and Halkier, 2018). A possible explanation for why the CYP79C specificities depended so strongly on the co-expressed biosynthetic pathways could be competition for available substrates. Although direct protein-protein interactions between CYP79s and the chain elongation machinery are unlikely, due to different subcellular localization (Sønderby et al., 2010), the variation may reflect the ability to obtain physical proximity between the CYP79s and the remaining core pathway enzymes in the heterologous tobacco host (Bassard and Halkier, 2018).

Our work describes a novel approach to investigate substrate specificities of CYP79 enzymes as compared to previous genetic and *in vitro* biochemical characterization (Wittstock and Halkier, 2002). For our two controls CYP79D2 and CYP79A2, our approach confirmed previous results, but also showed slightly expanded substrate specificities resulting in very minor additional GLS products (leucine accepted by CYP79D2 and tyrosine accepted by CYP79A2).

Interestingly, the anticipated outcome *a priori* of using either aliphatic or aromatic core pathways was not reflected in the results. Rather, both pathways were efficient in forming aromatic BGLS as well as aliphatic 2MP from CYP79C2, with even higher levels of 2MP being produced by the aromatic core pathway. Evidently, the distinction between aliphatic and aromatic core pathways—that is based on co-expression analysis (Wittstock and Halkier, 2002; Sønderby et al., 2010) of transcriptomics data from *A. thaliana*—is an oversimplification reflecting observations in the native host where many other factors including regulatory mechanisms may play a major role in controlling the biosynthetic machinery of glucosinolate formation and thus the observable glucosinolate profile. Apparently, the enzymes in the core pathways have substrate specificities toward both aliphatic and aromatic substrates when expressed in a heterologous host.

The main detected GLS products resulting from the two characterized CYP79C1 and CYP79C2 (BGLS, 1ME, 1MP, 2MP, 3MB, and 4MP) are not generally detected in *A. thaliana* Col-0 (Brown et al., 2003). Minor peaks tentatively identified by HPLC-UV as 1ME and 2MP have been detected in seeds of a few *A. thaliana* accessions, always at very low levels, and not from leaves of any accession (Kliebenstein et al., 2001). Neither was any corresponding hydrolysis product reported from 19 A*. thaliana* accessions (Hanschen et al., 2018). Two chain-elongated leucine-derived (3MB and 4MP) were claimed from unspecified *A. thaliana* accessions but precise experimental data were not presented (Reichelt et al., 2002). BGLS has repeatedly been referred to as present sporadically and at extremely low levels in *A. thaliana* (Windsor et al., 2005), but without presentation of actual data. In conclusion, BGLS, 1ME, 1MP, 2MP, 3MB, and 4MP have never been conclusively reported from any accession of *A. thaliana*. For this reason, the ability of CYP79C1 and CYP79C2 to channel phenylalanine, leucine and homologs (and to some degree valine and isoleucine) into GLS biosynthesis, is surprising. The specific expression in floral and embryonal tissues could suggest either a specific defensive function in these tissues or a role in non-defensive biochemistry.

Within the CYP79C subfamily, only CYP79C1 and CYP79C2 in *A. thaliana* have been annotated. The amino acid sequence of CYP79C1 shares 51.65% similarity with CYP79C2, although members in a subfamily have normally 55% amino acid sequence identity (Nelson, 2006). However, a slightly lower similarity is not uncommon since the rule is arbitrary and the decision to classify an enzyme into a subfamily depends on how it clusters within a phylogenetic tree and not strictly on the sequence similarity (Nelson, 2006). Furthermore, many reported RNA sequences share high percentage of identity (>70%) with the coding sequences of CYP79C1 and CYP79C2. These include many species from the Brassicaceae family and Cleomaceae family, both GLS-producing families in the Brassicales order.

The classification into P450 families and subfamilies is based on amino acid sequence and thus it is common to see enzymes with similar substrate specificity cluster in different subfamilies. This is apparent by CYP79C1 and CYP79C2 showing rather broad substrate specificities including both aliphatic and aromatic amino acids, which resembles what is observed for some of the CYP79Ds (**Table 1**) (Irmisch et al., 2013a; Irmisch et al., 2013b; Luck et al., 2016). Typically, the reported substrate specificity for CYP79s is toward single or related amino acids, and hence either toward aliphatic or aromatic amino acids. For instance, members of the CYP79B subfamily (e.g. CYP79B1, CYP79B2, and CYP79B3), channel tryptophan into GLS biosynthesis (**Table 1**) (Mikkelsen et al., 2000; Zhao et al., 2002; Naur et al., 2003). Similarly, CYP79E1 and CYP79E2 accept tyrosine (**Table 1**) (Nielsen and Møller, 2000). Likewise, the enzymes from CYP79A subfamily (CYP79A1, CYP79A2, CYP79A61) are reported to accept tyrosine, phenylalanine, or tryptophan, except for CYP79A118 that takes all three aromatic amino acids (**Table 1**) (Halkier et al., 1995; Wittstock and Halkier, 2000; Irmisch et al., 2015; Luck et al., 2017). The characterized members of the CYP79F subfamily has specificity toward chain-elongated methionine derivatives (**Table 1**) (Chen et al., 2003), except for CYP79F6 that has been proposed to metabolize homophenylalanine (Liu et al., 2016).

**Table 1:** The substrate specificity and affinity of the CYP79 family members.

CYP79 enzyme	Substrate specificity	Substrate affinity (K_M_)		Plant species	Locus		Reference
CYP79A1	Tyrosine	K_M,__=_ 220 µM		*Sorghum bicolor*	LOC8061413		Halkier et al., 1995
CYP79A2	Phenylalanine	K_M,__=_ 6.7 µM		*Arabidopsis thaliana*	AT5G05260		Wittstock and Halkier, 2000
CYP79A8	Leucine	Not tested		*Hordeum vulgare*	ACJ70085		Knoch et al., 2016
CYP79A12	Leucine	Not tested		*Hordeum vulgare*	ACM24114		Knoch et al., 2016
CYP79A61	Phenylalanine, Tryptophan	K_M,__Phe_ = 117.2 μM, K_M,__Trp_ = 150.2 µM		*Zea mays*	AKJ87843		Irmisch et al., 2015
CYP79A118 (N-terminal truncated)	Tyrosine, Phenylalanine, Tryptophan	K_M,__Tyr_ = 456 µM, K_M,__Phe_ = 21690 μM, K_M,__Trp_ = 24150 μM		*Taxus baccata*	ART92261		Luck et al., 2017
CYP79B1	Tryptophan	K_M,__=_ 29 µM		*Sinapis alba*	AAD03415		Naur et al., 2003
CYP79B2	Tryptophan	K_M,__=_ 21 µM		*Arabidopsis thaliana*	AT4G39950		Mikkelsen et al., 2000
CYP79B3	Tryptophan	Not tested		*Arabidopsis thaliana*	AT2G22330		Zhao et al., 2002
CYP79C1	Valine, Phenylalanine, Leucine, Isoleucine	Not tested Not tested Not tested Not tested		*Arabidopsis thaliana*	AT1G79370		This study
CYP79C2	Phenylalanine, Leucine, Isoleucine, Tryptophan, Tyrosine	Not tested Not tested Not tested Not tested Not tested		*Arabidopsis thaliana*	AT1G58260		This study
CYP79D1	Valine, Isoleucine	K_M,__Val_ = 2200 µM, K_M,__Ile_ = 1300 µM		*Manihot esculenta*	AAV97889		Andersen et al., 2000
CYP79D2	Valine, Isoleucine	Not tested Not tested		*Manihot esculenta*	AAV97888		Mikkelsen and Halkier, 2003
CYP79D3	Valine, Isoleucine	Not tested Not tested		*Lotus japonicus*	AAT11920		Forslund et al., 2004
CYP79D4	Valine, Isoleucine	Not tested Not tested		*Lotus japonicus*	AAT11921		Forslund et al., 2004
CYP79D6v3	Phenylalanine, Leucine, Isoleucine, Tryptophan, Tyrosine	K_M,__Phe_ = 744 μM, K_M,__Leu_ = 447 μM, K_M,__Ile_ = 526 µM, K_M,__Trp_ = 1427 μM, K_M,__Tyr_ = 1828 µM		*Populus trichocarpa*	AHF20912		Irmisch et al., 2013a
CYP79D6v4	Phenylalanine, Leucine, Isoleucine, Tryptophan, Tyrosine	Not tested Not tested Not tested Not tested Not tested		*Populus nigra*	AHI88992		Irmisch et al., 2013b
CYP79D7v2	Phenylalanine, Leucine, Isoleucine, Tryptophan	K_M,__Phe_ = 2901 μM, K_M,__Leu_ = 633 μM, K_M,__Ile_ = 851 µM, K_M,__Trp_ = 285 μM		*Populus trichocarpa*	AHF20913		Irmisch et al., 2013a
CYP79D60	Phenylalanine, Isoleucine, Leucine, Tryptophan, Tyrosine	K_M,__Phe_ = 580 μM, K_M,__Ile_ = 1280 μM, K_M,__Leu_ = 230 μM, K_M,__Trp_ = 2740 μM, K_M,__Tyr_ = 6090 μM,		*Erythroxylum fischeri*	AOW44273		Luck et al., 2016
CYP79D61	Phenylalanine, Isoleucine, Leucine, Tryptophan, Tyrosine	Not tested Not tested Not tested Not tested Not tested		*Erythroxylum fischeri*	AOW44271		Luck et al., 2016
CYP79D62	Phenylalanine, Isoleucine, Leucine, Tryptophan, Tyrosine	K_M,__Phe_ = 670 μM, K_M,__Ile_ = 3270 μM, K_M,__Leu_ = 590 μM, K_M,__Trp_ = 1090 μM, K_M,__Tyr_ = 4990 μM,		*Erythroxylum coca*	AOW44274		Luck et al., 2016
CYP79D63	Tryptophan	K_M,__=_ 480 µM		*Erythroxylum coca*	AOW4427		Luck et al., 2016
CYP79E1	Tyrosine	Not tested		*Triglochin maritima*	AF140609_1		Nielsen and Møller, 2000
CYP79E2	Tyrosine	Not tested		*Triglochin maritima*	AF140610_1		Nielsen and Møller, 2000
CYP79F1	1homoMet, 2homoMet, 3homoMet, 4homoMet, 5homoMet, 6homoMet,	Not tested K_M,__2homoMet_ = 34 µM, K_M,__3homoMet_ = 37 µM, K_M,__4homoMet_ = 194 µM, K_M,__5homoMet_ = 216 µM, K_M,__6homoMet_ = 74 µM		*Arabidopsis thaliana*	AT1G16410		Chen et al., 2003
CYP79F2	5homoMet, 6homoMet,	K_M,__5homoMet_ = 374 µM, K_M,__6homoMet_ = 26 µM	*Arabidopsis thaliana*			AT1G16400	Chen et al., 2003

1homoMet, homomethionine; 2homoMet, dihomomethionine; 3homoMet, trihomomethionine; 4homoMet, tetrahomomethionine; 5homoMet, pentahomomethionine; 6homoMet, hexahomomethionine.

Noticeably, CYP79F1 has affinity toward chain-elongated leucine derivatives when expressed in heterologous hosts such as *N. benthamiana* (Mikkelsen et al., 2010), which is beyond its endogenous enzymatic activity in wild type *A. thaliana*. A possible explanation to why some CYP79s exhibit an apparent different substrate specificity in heterologous hosts could be substrate availability or lack of co-factors such as e.g. chaperones (Fink, 1999). This raises the question of physiologically relevant substrate specificity versus promiscuity. The term enzyme promiscuity describes enzyme activities other than those for which an enzyme evolved and that are not part of the organism's physiology (Khersonsky and Tawfik, 2010). In summary, despite our present characterization of CYP79C1 and CYP79C2 in a heterologous host, further research is needed to reveal the biological role of CYP79C1 and CYP79C2 in *A. thaliana*.

Oximes are not only the intermediates in the biosynthesis of GLSs, but also involved in the biosynthesis of other defense compounds like cyanogenic glucosides, non-cyanogenic hydroxynitriles, and rhodiocyanosides (Møller and Conn, 1980; Andersen et al., 2000; Nielsen and Møller, 2000; Wittstock and Halkier, 2000; Forslund et al., 2004; Saito et al., 2012). Additionally, oximes are direct defense compounds, for example, in poplar (Irmisch et al., 2013a; Clavijo McCormick et al., 2014), and volatile aliphatic and aromatic oximes attract parasitoids when released after herbivory by caterpillars (Takabayashi et al., 1995; Zhang and Hartung, 2005; Wei et al., 2006). Moreover, volatile oximes are released as specific attractants for pollinators in moth-pollinated and night-blooming plants (Kaiser, 1993; Raguso, 2008; Vergara et al., 2011). Gaining knowledge about the function of the CYP79 enzymes provides molecular tools to engineer crop plants with new disease resistance properties (Brader et al., 2006).

In conclusion, characterization of CYP79 enzymes through pathway engineering in *N. benthamiana* is a powerful approach to assign biochemical function and substrate specificity to CYP79 enzymes, which is a prerequisite for understanding their functional role *in planta* and for using them as molecular tools in plant biotechnology to engineer glucosinolates and cyanogenic glucosides.

## Data Availability Statement

Publicly available datasets were analyzed in this study. This data can be found here: GSE113677, PRJEB24412.

## Author Contributions

CW, CC, and BH designed this study. CW, CC, BH, and NA interpreted results and wrote the manuscript based on a draft supplied by CW. CW was involved in all the experiments and supervised further cloning and infiltration experiments performed by MD. CC performed LC-MS method development and analysis. NA isolated and provided branched chain GLS standards for LC-MS analysis. The final manuscript was approved by all authors

## Funding

This work was supported by the Danish National Research Foundation (DNRF99) and the Novo Nordisk Fonden(NNF17OC0027710).

## Conflict of Interest

The authors declare that the research was conducted in the absence of any commercial or financial relationships that could be construed as a potential conflict of interest.

## Abbreviations

GLS, glucosinolate; BGLS, benzyl GLS; desulfo-GLS, dsGLS; 1ME, 1-methylethyl GLS; 2MP, 2-methylpropyl GLS; 1MP, 1-methylpropyl GLS; pOHB, *p*-hydroxybenzyl GLS; I3M, indol-3-ylmethyl GLS; 3MB, 3-methylbutyl GLS; 4MP, 4-methylpentyl GLS; 3MTP, 3-(methylthio)propyl GLS; 4MTB, 4-(methylthio)butyl GLS; 4MSB, 4-(methylsulfinyl)butyl GLS.

## References

[B1] Agerbirk N. Olsen C. E. Cipollini D. Ørgaard M. Linde-Laursen I. Chew F. S. (2014). Specific glucosinolate analysis reveals variable levels of epimeric glucobarbarins, dietary precursors of 5-phenyloxazolidine-2-thiones, in watercress types with contrasting chromosome numbers. J. Agric. Food Chem. 62, 9586–9596. 10.1021/jf5032795 25226408

[B2] Andersen M. D. Busk P. K. Svendsen I. (2000). Cytochromes P-450 from cassava (*Manihot esculenta* Crantz) catalyzing the first steps in the biosynthesis of the cyanogenic glucosides linamarin and lotaustralin. Cloning, functional expression in pichia pastoris, and substrate specificity of the isolated recombinant enzymes. J. Biol. Chem. 275, 1966–1975. 10.1074/jbc.275.3.1966 10636899

[B3] Bassard J.-E. Halkier B. A. (2018). How to prove the existence of metabolons? Phytochem. Rev. 17, 211–227. 10.1007/s11101-017-9509-1 29755303 PMC5932110

[B4] Blažević I. Montaut S. Burčul F. Olsen C. E. Burow M. Rollin P. (2020). Glucosinolate structural diversity, identification, chemical synthesis and metabolism in plants. Phytochemistry 169, 112100. 10.1016/j.phytochem.2019.112100 31771793

[B5] Brader G. Mikkelsen M. D. Halkier B. A. Tapio Palva E. (2006). Altering glucosinolate profiles modulates disease resistance in plants. Plant J. 46, 758–767. 10.1111/j.1365-313X.2006.02743.x 16709192

[B6] Brown P. D. Tokuhisa J. G. Reichelt M. Gershenzon J. (2003). Variation of glucosinolate accumulation among different organs and developmental stages of *Arabidopsis thaliana*. Phytochemistry 62, 471–481. 10.1016/S0031-9422(02)00549-6 12620360

[B7] Capovilla G. Delhomme N. Collani S. Shutava I. Bezrukov I. Symeonidi E. (2018). PORCUPINE regulates development in response to temperature through alternative splicing. Nat. Plants 4, 534–539. 10.1038/s41477-018-0176-z 29988152

[B8] Chen S. Glawischnig E. Jørgensen K. Naur P. Jørgensen B. Olsen C.-E. (2003). CYP79F1 and CYP79F2 have distinct functions in the biosynthesis of aliphatic glucosinolates in *Arabidopsis*. Plant J. 33, 923–937. 10.1046/j.1365-313X.2003.01679.x 12609033

[B9] Clavijo McCormick A. Irmisch S. Reinecke A. Boeckler G. A. Veit D. Reichelt M. (2014). Herbivore-induced volatile emission in black poplar: regulation and role in attracting herbivore enemies. Plant Cell Environ. 37, 1909–1923. 10.1111/pce.12287 24471487

[B10] Crocoll C. Halkier B. A. Burow M. (2016a). Analysis and quantification of glucosinolates. Curr. Protoc. Plant Biol. 1, 385–409. 10.1002/cppb.20027 30775863

[B11] Crocoll C. Mirza N. Reichelt M. Gershenzon J. Halkier B. A. (2016b). Optimization of Engineered production of the glucoraphanin Precursor Dihomomethionine in *Nicotiana benthamiana*. Front. Bioeng. Biotechnol. 4, 14. 10.3389/fbioe.2016.00014 26909347 PMC4754535

[B12] Fink A. L. (1999). Chaperone-mediated protein folding. Physiol. Rev. 79, 425–449. 10.1152/physrev.1999.79.2.425 10221986

[B13] Forslund K. Morant M. Jørgensen B. Olsen C. E. Asamizu E. Sato S. (2004). Biosynthesis of the nitrile glucosides rhodiocyanoside A and D and the cyanogenic glucosides lotaustralin and linamarin in *Lotus japonicus*. Plant Physiol. 135, 71–84. 10.1104/pp.103.038059 15122013 PMC429334

[B14] Geu-Flores F. Nour-Eldin H. H. Nielsen M. T. Halkier B. A. (2007). USER fusion: a rapid and efficient method for simultaneous fusion and cloning of multiple PCR products. Nucleic Acids Res. 35, e55. 10.1093/nar/gkm106 17389646 PMC1874642

[B15] Geu-Flores F. Nielsen M. T. Nafisi M. Møldrup M. E. Olsen C. E. Motawia M. S. (2009). Glucosinolate engineering identifies a gamma-glutamyl peptidase. Nat. Chem. Biol. 5, 575–577. 10.1038/nchembio.185 19483696

[B16] Halkier B. A. Gershenzon J. (2006). Biology and biochemistry of glucosinolates. Annu. Rev. Plant Biol. 57, 303–333. 10.1146/annurev.arplant.57.032905.105228 16669764

[B17] Halkier B. A. Nielsen H. L. Koch B. Møller B. L. (1995). Purification and characterization of recombinant cytochrome P450TYR expressed at high levels in *Escherichia coli*. Arch. Biochem. Biophys. 322, 369–377. 10.1006/abbi.1995.1477 7574710

[B18] Hanschen F. S. Pfitzmann M. Witzel K. Stützel H. Schreiner M. Zrenner R. (2018). Differences in the enzymatic hydrolysis of glucosinolates increase the defense metabolite diversity in 19 *Arabidopsis thaliana* accessions. Plant Physiol. Biochem. 124, 126–135. 10.1016/j.plaphy.2018.01.009 29366972

[B19] Irmisch S. McCormick A. C. Boeckler G. A. Schmidt A. Reichelt M. Schneider B. (2013a). Two herbivore-induced cytochrome P450 enzymes CYP79D6 and CYP79D7 catalyze the formation of volatile aldoximes involved in poplar defense. Plant Cell 25, 4737–4754. 10.1105/tpc.113.118265 24220631 PMC3875747

[B20] Irmisch S. Unsicker S. B. Gershenzon J. Köllner T. G. (2013b). Identification and characterization of CYP79D6v4, a cytochrome P450 enzyme producing aldoximes in black poplar (*Populus nigra*). Plant Signal. Behav. 8, e27640. 10.4161/psb.27640 24390071 PMC4091388

[B21] Irmisch S. Zeltner P. Handrick V. Gershenzon J. Köllner T. G. (2015). The maize cytochrome P450 CYP79A61 produces phenylacetaldoxime and indole-3-acetaldoxime in heterologous systems and might contribute to plant defense and auxin formation. BMC Plant Biol. 15, 128. 10.1186/s12870-015-0526-1 26017568 PMC4446944

[B22] Jensen L. M. Kliebenstein D. J. Burow M. (2015). Investigation of the multifunctional gene AOP3 expands the regulatory network fine-tuning glucosinolate production in *Arabidopsis*. Front. Plant Sci. 6, 762. 10.3389/fpls.2015.00762 26442075 PMC4585220

[B23] Kaiser R. A. J. (1993). “On the scent of orchids,” in Bioactive Volatile Compounds from Plants. Eds. Teranishi R. Buttery R. G. Sugisawa H. (San Francisco, California: American Chemical Society), 240–268. 10.1021/bk-1993-0525.ch018

[B24] Khersonsky O. Tawfik D. S. (2010). Enzyme promiscuity: a mechanistic and evolutionary perspective. Annu. Rev. Biochem. 79, 471–505. 10.1146/annurev-biochem-030409-143718 20235827

[B25] Klepikova A. V. Kasianov A. S. Gerasimov E. S. Logacheva M. D. Penin A. A. (2016). A high resolution map of the *Arabidopsis thaliana* developmental transcriptome based on RNA-seq profiling. Plant J. 88, 1058–1070. 10.1111/tpj.13312 27549386

[B26] Kliebenstein D. J. Kroymann J. Brown P. Figuth A. Pedersen D. Gershenzon J. (2001). Genetic control of natural variation in Arabidopsis glucosinolate accumulation. Plant Physiol. 126, 811–825. 10.1104/pp.126.2.811 11402209 PMC111171

[B27] Knoch E. Motawie M. S. Olsen C. E. Møller B. L. Lyngkjaer M. F. (2016). Biosynthesis of the leucine derived α-, β- and γ-hydroxynitrile glucosides in barley (*Hordeum vulgare* L.). Plant J. 88, 247–256. 10.1111/tpj.13247 27337134

[B28] \Liu T. Zhang X. Yang H. Agerbirk N. Qiu Y. Wang H. (2016). Aromatic glucosinolate biosynthesis pathway in *Barbarea vulgaris* and its response to *Plutella xylostella* infestation. Front. Plant Sci. 7, 83. 10.3389/fpls.2016.00083 26904055 PMC4744896

[B29] Luck K. Jirschitzka J. Irmisch S. Huber M. Gershenzon J. Köllner T. G. (2016). CYP79D enzymes contribute to jasmonic acid-induced formation of aldoximes and other nitrogenous volatiles in two *Erythroxylum* species. BMC Plant Biol. 16. 10.1186/s12870-016-0910-5 PMC5050915 27716065

[B30] Luck K. Jia Q. Huber M. Handrick V. Wong G. K.-S. Nelson D. R. (2017). CYP79 P450 monooxygenases in gymnosperms: CYP79A118 is associated with the formation of taxiphyllin in *Taxus baccata*. Plant Mol. Biol. 95, 169–180. 10.1007/s11103-017-0646-0 28795267 PMC5594043

[B31] Møldrup M. E. Geu-Flores F. Olsen C. E. Halkier B. A. (2011). Modulation of sulfur metabolism enables efficient glucosinolate engineering. BMC Biotechnol. 11, 12. 10.1186/1472-6750-11-12 21281472 PMC3042935

[B32] Møller B. L. Conn E. E. (1980). The biosynthesis of cyanogenic glucosides in higher plants. Channeling of intermediates in dhurrin biosynthesis by a microsomal system from *Sorghum bicolor* (Linn) Moench. J. Biol. Chem. 255, 3049–3056. 7358727

[B33] Mikkelsen M. D. Halkier B. A. (2003). Metabolic engineering of valine- and isoleucine-derived glucosinolates in *Arabidopsis* expressing CYP79D2 from Cassava. Plant Physiol. 131, 773–779. 10.1104/pp.013425 12586901 PMC166853

[B34] Mikkelsen M. D. Hansen C. H. Wittstock U. Halkier B. A. (2000). Cytochrome P450 CYP79B2 from *Arabidopsis* catalyzes the conversion of tryptophan to indole-3-acetaldoxime, a precursor of indole glucosinolates and indole-3-acetic acid. J. Biol. Chem. 275, 33712–33717. 10.1074/jbc.M001667200 10922360

[B35] Mikkelsen M. D. Olsen C. E. Halkier B. A. (2010). Production of the cancer-preventive glucoraphanin in tobacco. Mol. Plant 3, 751–759. 10.1093/mp/ssq020 20457641

[B36] Naur P. Hansen C. H. Bak S. Hansen B. G. Jensen N. B. Nielsen H. L. (2003). CYP79B1 from *Sinapis alba* converts tryptophan to indole-3-acetaldoxime. Arch. Biochem. Biophys. 409, 235–241. 10.1016/S0003-9861(02)00567-2 12464264

[B37] Nelson D. R. (2006). Cytochrome P450 nomenclature, 2004. Methods Mol. Biol. 320, 1–10. 10.1385/1-59259-998-2:1 16719369

[B38] Nielsen J. S. Møller B. L. (2000). Cloning and expression of cytochrome P450 enzymes catalyzing the conversion of tyrosine to *p*-hydroxyphenylacetaldoxime in the biosynthesis of cyanogenic glucosides in *Triglochin maritima*. Plant Physiol. 122, 1311–1322. 10.1104/pp.122.4.1311 10759528 PMC58967

[B39] Nielsen M. Ard R. Leng X. Ivanov M. Kindgren P. Pelechano V. (2019). Transcription-driven chromatin repression of intragenic transcription start sites. PloS Genet. 15. 10.1371/journal.pgen.1007969 PMC6373976 30707695

[B40] Nour-Eldin H. H. Hansen B. G. Nørholm M. H. H. Jensen J. K. Halkier B. A. (2006). Advancing uracil-excision based cloning towards an ideal technique for cloning PCR fragments. Nucleic Acids Res. 34, e122. 10.1093/nar/gkl635 17000637 PMC1635280

[B41] Olsen C. E. Huang X.-C. Hansen C. I. C. Cipollini D. Ørgaard M. Matthes A. (2016). Glucosinolate diversity within a phylogenetic framework of the tribe Cardamineae (Brassicaceae) unraveled with HPLC-MS/MS and NMR-based analytical distinction of 70 desulfoglucosinolates. Phytochemistry 132, 33–56. 10.1016/j.phytochem.2016.09.013 27743600

[B42] Petersen A. Crocoll C. Halkier B. A. (2019a). De novo production of benzyl glucosinolate in *Escherichia coli*. Metab. Eng. 54, 24–34. 10.1016/j.ymben.2019.02.004 30831267

[B43] Petersen A. Hansen L. G. Mirza N. Crocoll C. Mirza O. Halkier B. A. (2019b). Changing substrate specificity and iteration of amino acid chain elongation in glucosinolate biosynthesis through targeted mutagenesis of *Arabidopsis* methylthioalkylmalate synthase 1. Biosci. Rep. 39, BSR20190446. 10.1042/BSR20190446 31175145 PMC6603273

[B44] Raguso R. A. (2008). Wake up and smell the roses: the ecology and evolution of floral scent. Annu. Rev. Ecol. Evol. Syst. 39, 549–569. 10.1146/annurev.ecolsys.38.091206.095601

[B45] Reichelt M. Brown P. D. Schneider B. Oldham N. J. Stauber E. Tokuhisa J. (2002). Benzoic acid glucosinolate esters and other glucosinolates from *Arabidopsis thaliana*. Phytochemistry 59, 663–671. 10.1016/S0031-9422(02)00014-6 11867099

[B46] Sønderby I. E. Geu-Flores F. Halkier B. A. (2010). Biosynthesis of glucosinolates–gene discovery and beyond. Trends Plant Sci. 15, 283–290. 10.1016/j.tplants.2010.02.005 20303821

[B47] Saito S. Motawia M. S. Olsen C. E. Møller B. L. Bak S. (2012). Biosynthesis of rhodiocyanosides in *Lotus japonicus*: rhodiocyanoside A is synthesized from (Z)-2-methylbutanaloxime *via* 2-methyl-2-butenenitrile. Phytochemistry 77, 260–267. 10.1016/j.phytochem.2012.01.020 22385904

[B48] Schmid M. W. Schmidt A. Klostermeier U. C. Barann M. Rosenstiel P. Grossniklaus U. (2012). A powerful method for transcriptional profiling of specific cell types in eukaryotes: laser-assisted microdissection and RNA sequencing. PloS One 7. 10.1371/journal.pone.0029685 PMC3266888 22291893

[B49] Takabayashi J. Takahashi S. Dicke M. Posthumus M. A. (1995). Developmental stage of herbivore *Pseudaletia separata* affects production of herbivore-induced synomone by corn plants. J. Chem. Ecol. 21, 273–287. 10.1007/BF02036717 24234060

[B50] Vergara R. C. Torres-Araneda A. Villagra D. A. (2011). Are eavesdroppers multimodal? Sensory exploitation of floral signals by a non-native cockroach *Blatta orientalis*. Curr. Zool. 57, 162–174. 10.1093/czoolo/57.2.162 PMC8488991 34617935

[B51] Voinnet O. Rivas S. Mestre P. Baulcombe D. (2003). Retracted: an enhanced transient expression system in plants based on suppression of gene silencing by the p19 protein of tomato bushy stunt virus. Plant J. 33, 949–956. 10.1046/j.1365-313X.2003.01676.x 12609035

[B52] Wei J. N. Zhu J. Kang L. (2006). Volatiles released from bean plants in response to agromyzid flies. Planta 224, 279–287. 10.1007/s00425-005-0212-x 16404576

[B53] Windsor A. J. Reichelt M. Figuth A. Svatos A. Kroymann J. Kliebenstein D. J. (2005). Geographic and evolutionary diversification of glucosinolates among near relatives of *Arabidopsis thaliana* (Brassicaceae). Phytochemistry 66, 1321–1333. 10.1016/j.phytochem.2005.04.016 15913672

[B54] Wittstock U. Halkier B. A. (2000). Cytochrome P450 CYP79A2 from *Arabidopsis thaliana* L. catalyzes the conversion of L-phenylalanine to phenylacetaldoxime in the biosynthesis of benzylglucosinolate. J. Biol. Chem. 275, 14659–14666. 10.1074/jbc.275.19.14659 10799553

[B55] Wittstock U. Halkier B. A. (2002). Glucosinolate research in the *Arabidopsis* era. Trends Plant Sci. 7, 263–270. 10.1016/S1360-1385(02)02273-2 12049923

[B56] Zhang A. Hartung J. S. (2005). Phenylacetaldehyde O-methyloxime: a volatile compound produced by grapefruit leaves infected with the citrus canker pathogen, *Xanthomonas axonopodis* pv. *citri*. J. Agric. Food Chem. 53, 5134–5137. 10.1021/jf050533x 15969487

[B57] Zhao Y. Hull A. K. Gupta N. R. Goss K. A. Alonso J. Ecker J. R. (2002). Trp-dependent auxin biosynthesis in *Arabidopsis*: involvement of cytochrome P450s CYP79B2 and CYP79B3. Genes Dev. 16, 3100–3112. 10.1101/gad.1035402 12464638 PMC187496

