# Supplementary material for: Characterization of Arabidopsis CYP79C1 and CYP79C2 by Glucosinolate Pathway Engineering in Nicotiana benthamiana Shows Substrate Specificity Toward a Range of Aliphatic and Aromatic Amino Acids
Source: Front Plant Sci. 2020 Feb 14;11:57. doi: 10.3389/fpls.2020.00057 (PMC7033466; doi:10.3389/fpls.2020.00057)

Supplementary figures and methods for:

Title: Characterization of Arabidopsis CYP79C1 and CYP79C2 by Glucosinolate Pathway Engineering in *Nicotiana benthamiana* Shows Substrate Specificity Toward a Range of Aliphatic and Aromatic Amino Acids

Authors: Cuiwei Wang, Mads Møller Dissing, Niels Agerbirk, Christoph Crocoll and Barbara Ann Halkier

Journal: Frontiers in Plant Science

Contents:

Supplementary Methods. Identification of glucosinolates and desulfo-glucosinolates in plant extracts by LC-MS/Q-TOF.

Supplementary Figure S1. Spectroscopic data of authentic 1ME (potassium 1-methyl-ethylglucosinolate) isolated from *Tropaeolum peregrinum* seeds.

Supplementary Figure S2. Extracted Ion Chromatograms (EIC) of valine-, leucine- and isoleucine-derived intact GLSs (1ME, 2MP and 1MP, respectively) in extracts from *N. benthamiana* after co-expression of CYP79s with the aliphatic core pathway.

Supplementary Figure S3. Extracted Ion Chromatograms (EIC) of phenylalanine-derived intact GLS (BGLS) in extracts from *N. benthamiana* after co-expression of CYP79s with the aliphatic core pathway.

Supplementary Figure S4. Extracted Ion Chromatograms (EIC) of tyrosine and tryptophan-derived intact GLSs (pOHB and I3M, respectively) in extracts from *N. benthamiana* after co-expression of CYP79s with the aliphatic core pathway.

Supplementary Figure S5. Extracted Ion Chromatograms (EIC) of valine-, leucine- and isoleucine-derived intact GLSs (1ME, 2MP and 1MP, respectively) in extracts from *N. benthamiana* after co-expression of CYP79s with the aromatic core pathway.

Supplementary Figure S6. Extracted Ion Chromatograms (EIC) of phenylalanine-derived intact GLS (BGLS) in extracts from *N. benthamiana* after co-expression of CYP79s with the aromatic core pathway.

Supplementary Figure S7. Extracted Ion Chromatograms (EIC) of tyrosine and tryptophan-derived intact GLSs (pOHB and I3M, respectively) in extracts from *N. benthamiana* after co-expression of CYP79s with the aromatic core pathway.

Supplementary Figure S8. LC-MS/QqQ chromatograms traces of valine-derived desulfo-GLS (1ME) in extracts from *N. benthamiana* after co-expression of CYP79s with the aliphatic core pathway.

Supplementary Methods. Identification of glucosinolates and desulfo-glucosinolates in plant extracts by LC-MS/Q-TOF

Metabolomics on plant extracts of infiltrated *N. benthamiana* leaves was performed by LC-MS/Q-TOF. Separation was achieved on a Dionex UltiMate 3000 Quaternary Rapid Separation UHPLC+ focused system (Thermo Fisher Scientific, Germering, Germany) equipped with a Kinetex 1.7 µm XB-C18 column (150 × 2.1 mm, 1.7 µm, 100 Å, Phenomenex). For eluting 0.05% (v/v) formic acid in H_2O and MeCN [supplied with 0.05% (v/v) formic acid] were employed as mobile phases A and B, respectively. Gradient conditions were as follows: 0.0−14.0 min 2−20% B; 14.0−20.0 min 20−45% B, 20.0−24.5 min 45−100% B, 24.5−26.5 min 100% B, 26.5−26.55 min 100−2% B, and 26.55−30.0 min 2% B. The flow rate of the mobile phase was 300 µL/min. The column temperature was maintained at 30°C. UV chromatograms were acquired at 229, 260, 310, and 345 nm. The UHPLC was coupled to a Compact micrOTOF-Q mass spectrometer (Bruker, Bremen, Germany) equipped with an electrospray ion source (ESI) operated in positive or negative ionization mode. The ion spray voltage was maintained at +4500 V or -3900 V in positive and negative ionization mode, respectively. The dry temperature was set to 250°C, and the dry gas flow was set to 8 L/min. Nitrogen was used as the dry gas, nebulizing gas, and collision gas. The nebulizing gas was set to 2.5 bar and collision energy to 15 eV. HRESIMS and MS/MS spectra were acquired in an *m/z* range from 50 to 1000 amu at a sampling rate of 2 Hz. Sodium formate clusters were used for mass calibration. All files were automatically calibrated by postprocessing. In the case of 1-methylethyl GLS (1ME), BGLS, *p*-hydroxybenzyl GLS (pOHB), and indol-3-ylmethyl GLS (I3M), native GLS identification was by retention time, accurate mass and MS/MS comparison with authentic standards. In the case of 2-methylpropyl GLS (2MP) and 1-methylpropyl GLS (1MP), authentic standards of native GLSs were not available, but retention time and MS parameters were similarly detected. The side chain structures of the latter were identified conclusively using authentic dsGLSs (**Supplementary Table S2**). Authentic standards of BGLS, pOHB and I3M were purchased from PhytoLab, Vestenbergsgreuth, Germany. Authentic 1ME (potassium isopropylglucosinolate) was isolated from seeds of *Tropaeolum peregrinum* L. (Chiltern Seeds, Ulverston, UK) using a previously described protocol including isolation of pooled intact GLSs by salt elution of a DEAE-Sephadex column followed by desalting and preparative HPLC of intact GLSs using CF_3COOH in the HPLC eluents (Agerbirk et al., 2014). The 1ME resulting from this protocol was contaminated with potassium acetate, and was desalted by a final HPLC without acid added to the eluents, lyophilized and subjected to ^{1}H NMR, ^{13}C NMR and ion trap LC-MS (Agerbirk et al., 2014). Chromatogram, NMR spectra and MS of the isolated intact 1ME are shown as **Supplementary Figure S1**. The NMR data for 1ME essentially agreed with published data (Montaut et al., 2010).

Montaut, S., Bleeker, R. S., Jacques, C., 2010b. Phytochemical constituents of *Cardamine diphylla*. Can. J. Chem. 88, 50-55.

Supplementary Figure S1. Spectroscopic data of authentic 1ME (potassium 1-methyl-ethyl glucosinolate) isolated from *Tropaeolum peregrinum* seeds. **A**: ^{1}H NMR (400 MHz, solvent D_2O, internal std. dioxane at 3.75 ppm). **B**: Same spectrum but focused. **C**: ^{13}C NMR of the same sample (100 MHz, solvent D_2O, internal std. dioxane at 67.400 ppm). **D**: DEPT135 of the same sample for distinction of carbon types. **E**. Ion trap HPLC-MS (negative mode) of the glucosinolate, Eluents acidified (HCOOH) and spiked with NaCl.

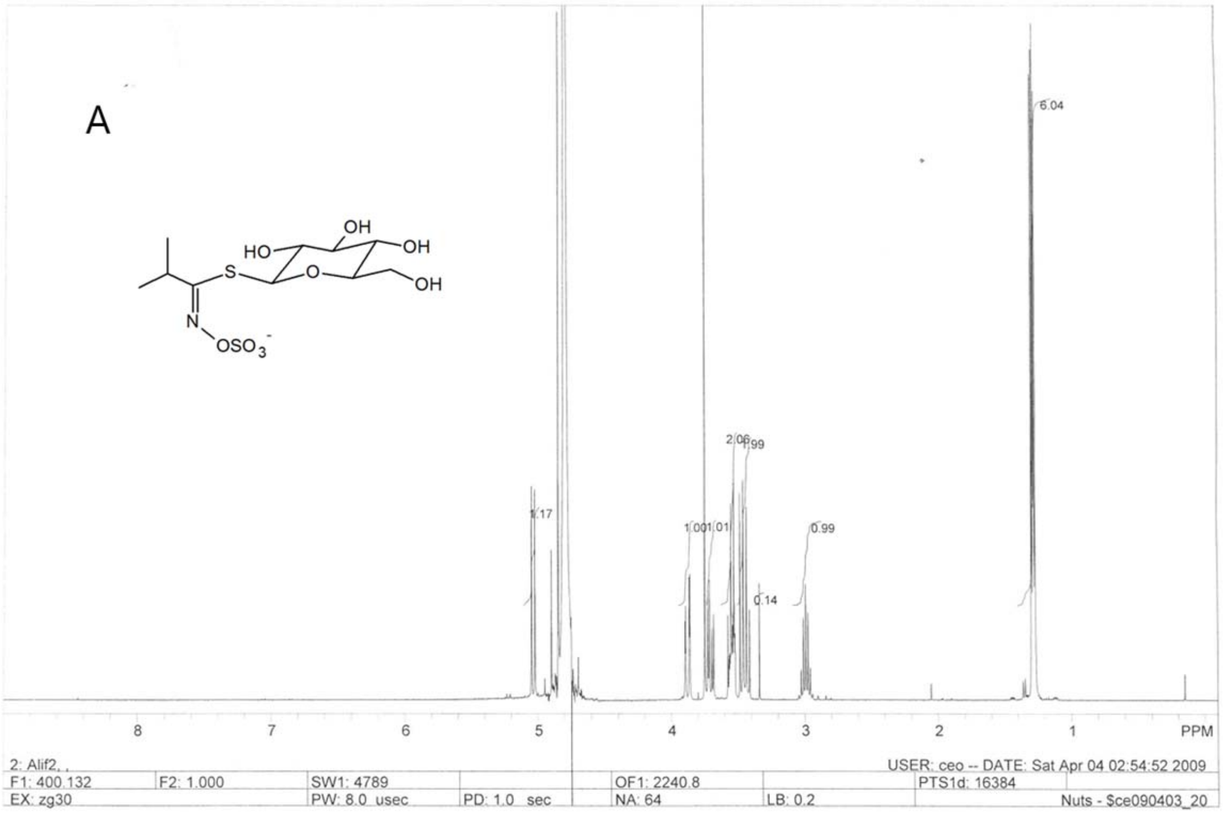

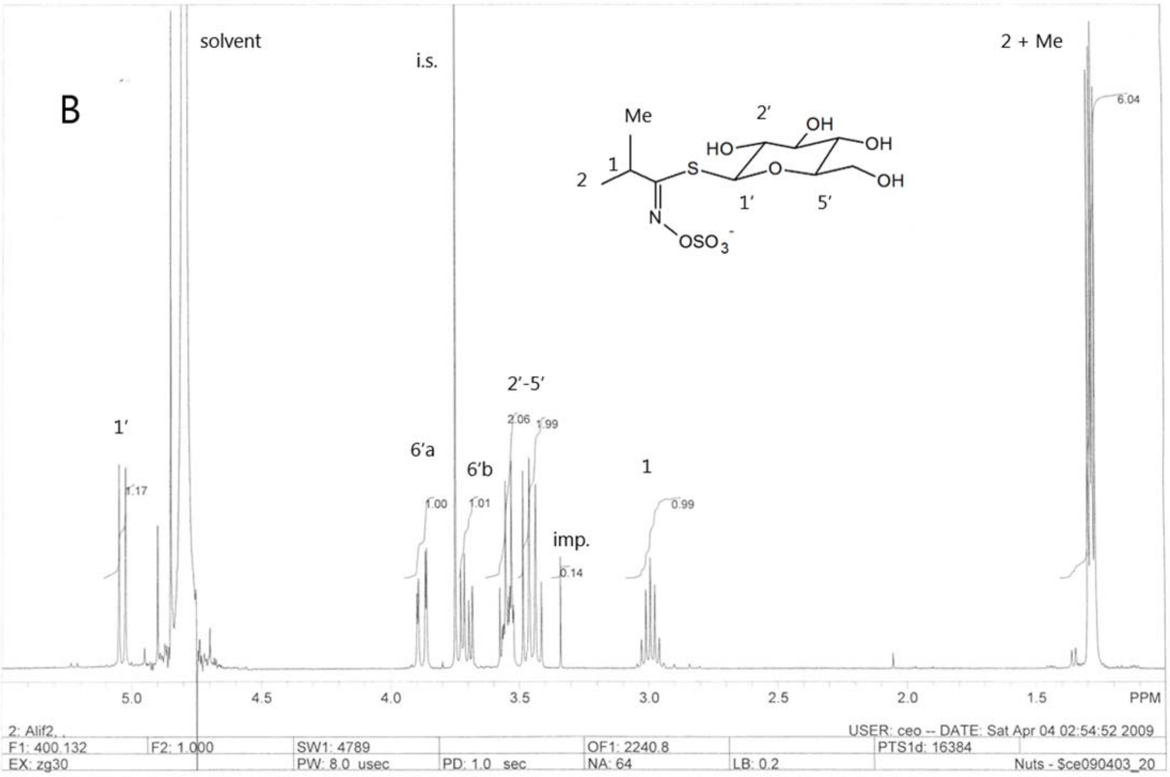
B
solvent
i.s.
2 + Me
Me
2'
HO
OH
OH
OH
S
O
1
2
1'
5'
N
OSO_3^-
6.04
2'-5'
1'
6'a
6'b
1
imp.
1.17
1.00
1.01
2.06
1.99
0.99
0.14
5.0
4.5
4.0
3.5
3.0
2.5
2.0
1.5
PPM
2: Alif2, ,
USER: ceo -- DATE: Sat Apr 04 02:54:52 2009
F1: 400.132
F2: 1.000
SW1: 4789
OF1: 2240.8
PTS1d: 16384
EX: zg30
PW: 8.0 usec
PD: 1.0 sec
NA: 64
LB: 0.2
Nuts - $ce090403_20

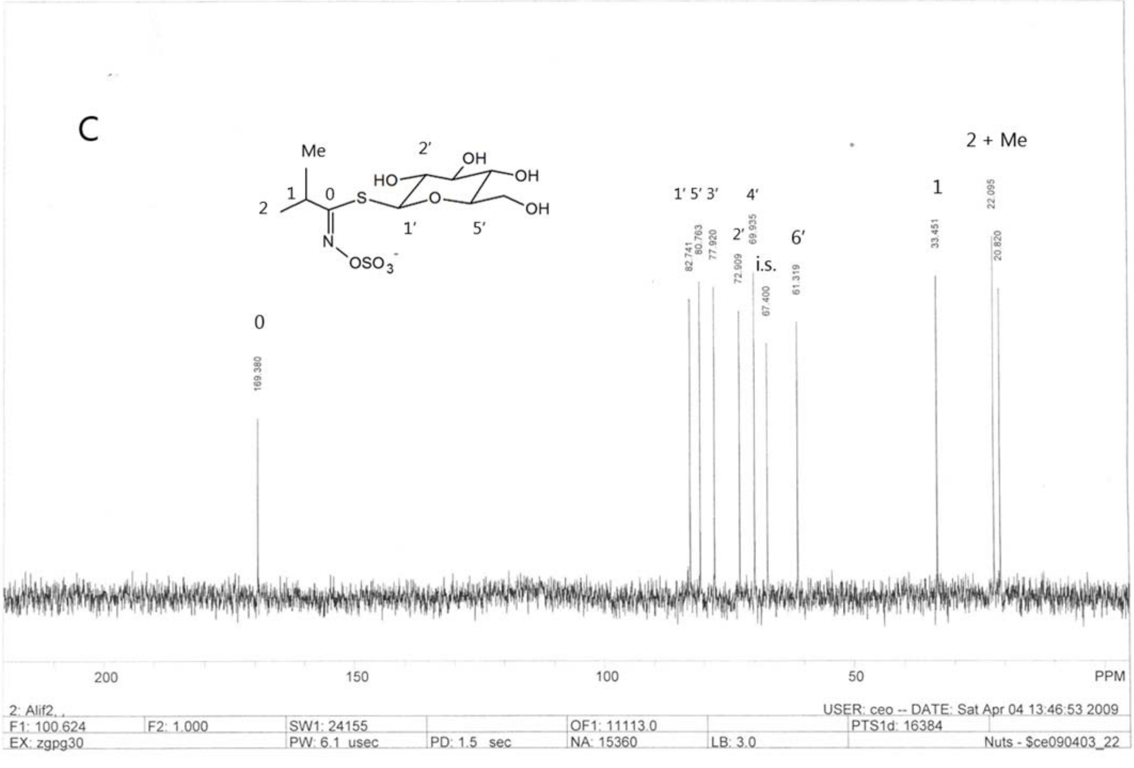
C
Me
2'
HO
OH
OH
OH
S
O
2
1
0
1'
5'
N
OSO_3^-
2 + Me
1' 5' 3'
4'
2'
i.s.
6'
1
0
169.380
82.741
80.763
77.920
72.909
69.935
67.400
61.319
33.451
22.095
20.820
200
150
100
50
PPM
2: Alif2, ,
USER: ceo -- DATE: Sat Apr 04 13:46:53 2009
F1: 100.624
F2: 1.000
SW1: 24155
OF1: 11113.0
PTS1d: 16384
EX: zgpg30
PW: 6.1 usec
PD: 1.5 sec
NA: 15360
LB: 3.0
Nuts - $ce090403_22

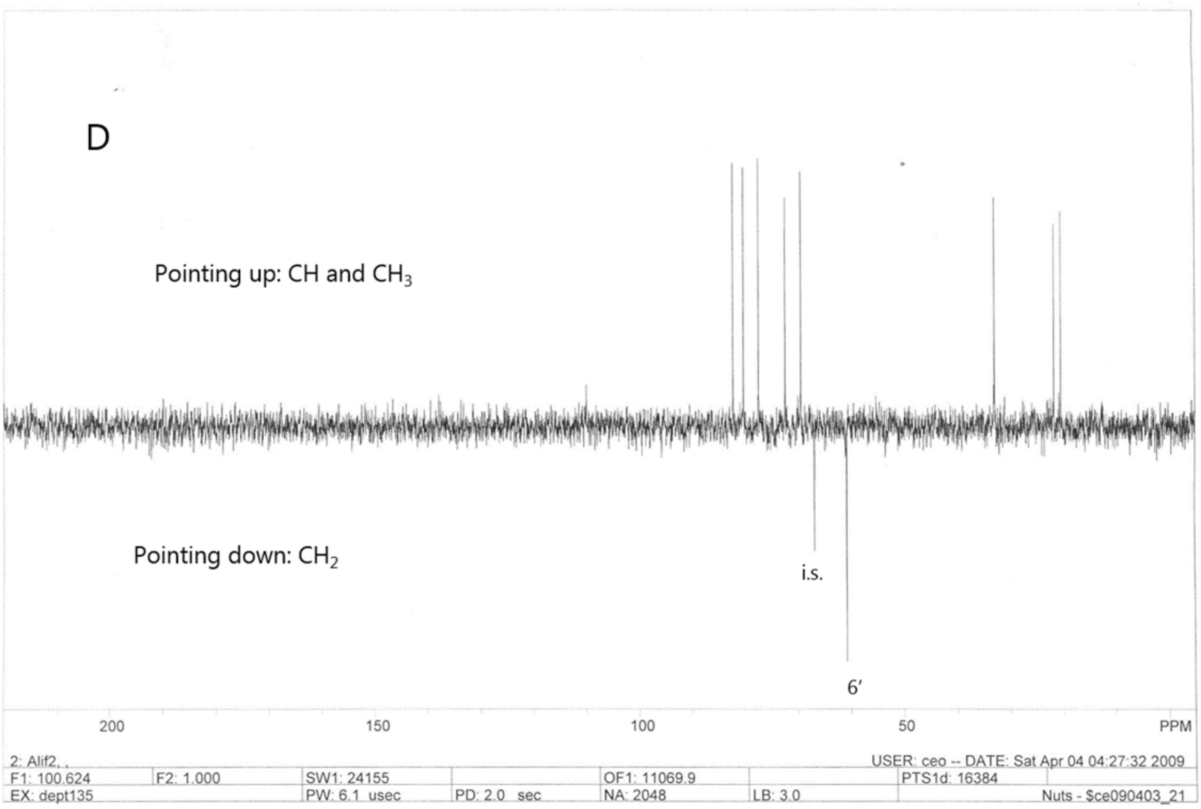
D
Pointing up: CH and CH3
Pointing down: CH2
i.s.
6′
200
150
100
50
PPM
2: Alif2, ,
F1: 100.624
F2: 1.000
SW1: 24155
OF1: 11069.9
USER: ceo -- DATE: Sat Apr 04 04:27:32 2009
PTS1d: 16384
EX: dept135
PW: 6.1 usec
PD: 2.0 sec
NA: 2048
LB: 3.0
Nuts - $ce090403_21

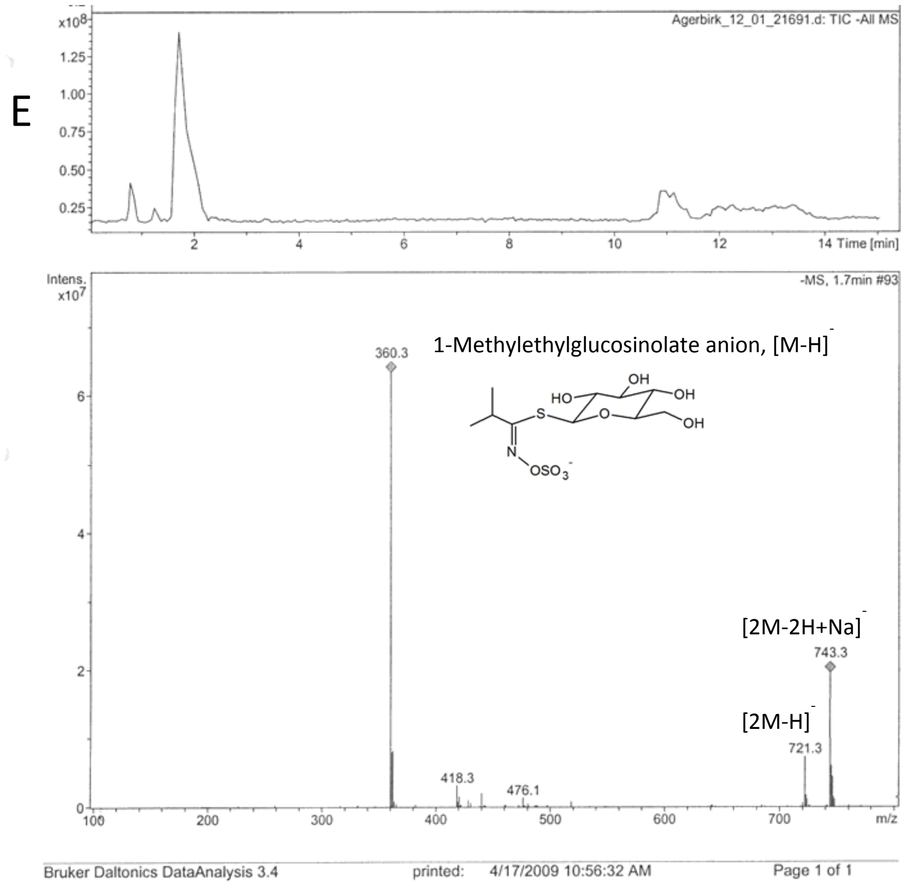
E
Agerbirk_12_01_21691.d: TIC -All MS
x10^8
Time [min]
Intens. x10^7
-MS, 1.7min #93
360.3
1-Methylethylglucosinolate anion, [M-H]-
HO
OH
OH
OH
S
O
N
OSO3-
[2M-2H+Na]-
743.3
[2M-H]-
721.3
418.3
476.1
m/z
Bruker Daltonics DataAnalysis 3.4
printed: 4/17/2009 10:56:32 AM
Page 1 of 1

Supplementary Figure S2. Extracted Ion Chromatograms (EIC) of valine-, leucine- and isoleucine-derived intact GLSs (1ME, 2MP and 1MP, respectively) in extracts from *N. benthamiana* after co-expression of CYP79s with the aliphatic core pathway. **(A)** Reference standard for 1ME. **(B)** p19 infiltration control. **(C)** Negative control of the aliphatic core pathway without any CYP79 co-expression. **(D)** Positive control of CYP79D2 co-expressed with the aliphatic core pathway. **(E)** CYP79C1 co-expressed with the aliphatic core pathway and **(F)** CYP79C2 co-expressed with the aliphatic core pathway.

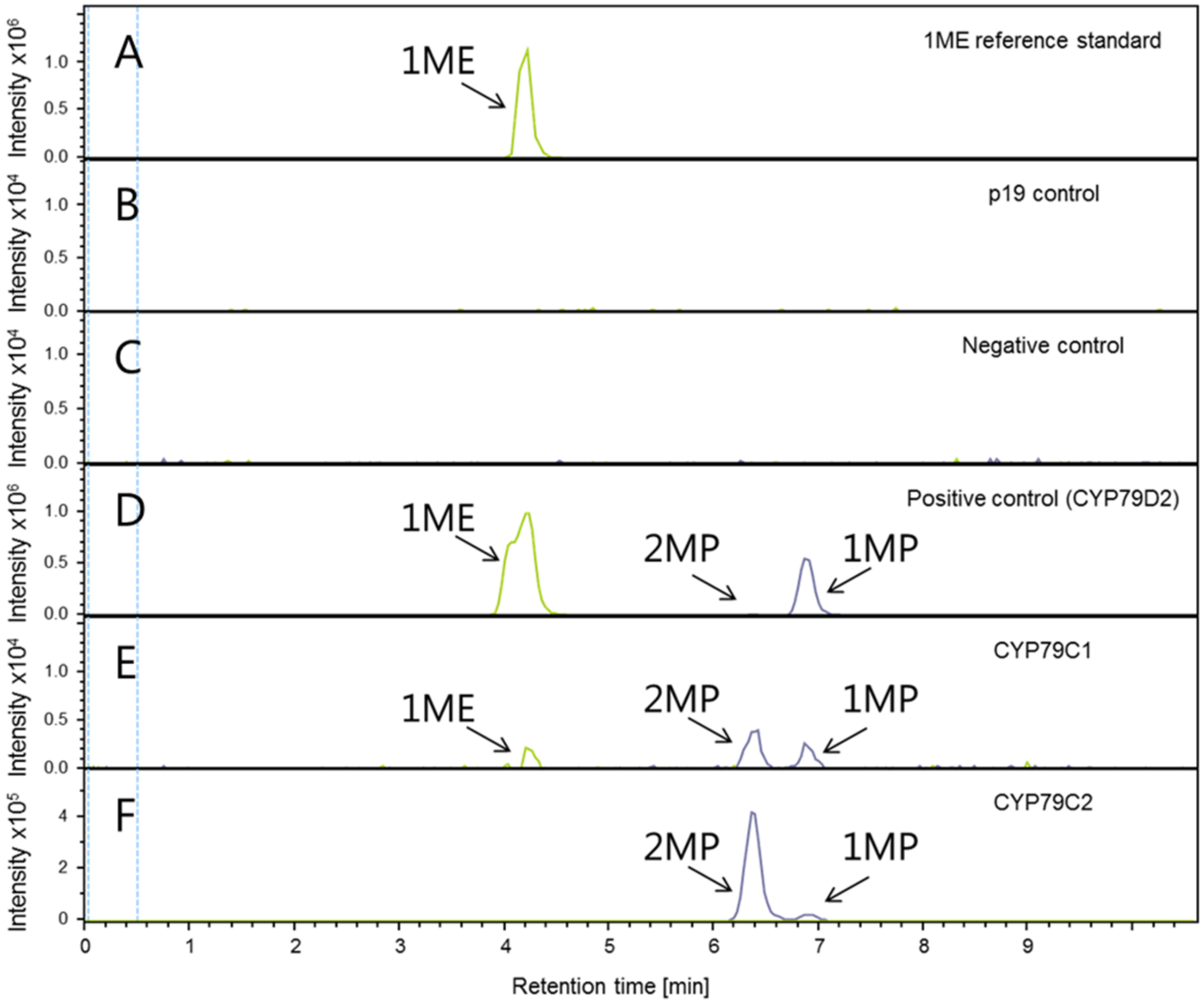

Supplementary Figure S3. Extracted Ion Chromatograms (EIC) of phenylalanine-derived intact GLS (BGLS) in extracts from *N. benthamiana* after co-expression of CYP79s with the aliphatic core pathway. **(A)** Reference standard for BGLS. **(B)** p19 infiltration control. **(C)** Negative control of the aliphatic core pathway without any CYP79 co-expression. **(D)** Positive control of CYP79D2 co-expressed with the aliphatic core pathway. **(E)** CYP79C1 co-expressed with the aliphatic core pathway and **(F)** CYP79C2 co-expressed with the aliphatic core pathway.

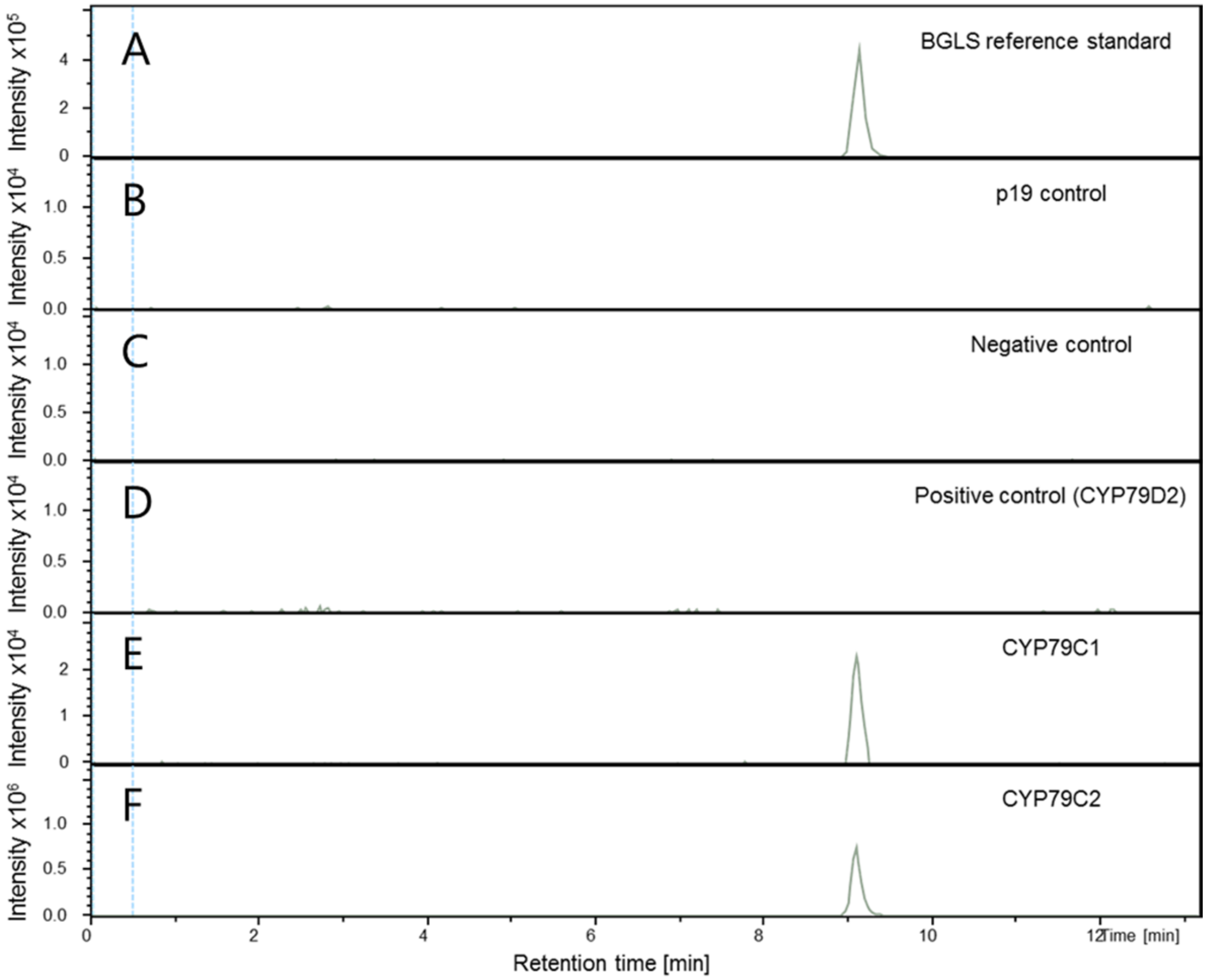

Supplementary Figure S4. Extracted Ion Chromatograms (EIC) of tyrosine and tryptophan-derived intact GLSs (pOHB and I3M, respectively) in extracts from *N. benthamiana* after co-expression of CYP79s with the aliphatic core pathway. **(A)** Reference standard for pOHB and I3M. **(B)** p19 infiltration control. **(C)** Negative control of the aliphatic core pathway without any CYP79 co-expression. **(D)** Positive control of CYP79D2 co-expressed with the aliphatic core pathway. **(E)** CYP79C1 co-expressed with the aliphatic core pathway and **(F)** CYP79C2 co-expressed with the aliphatic core pathway.

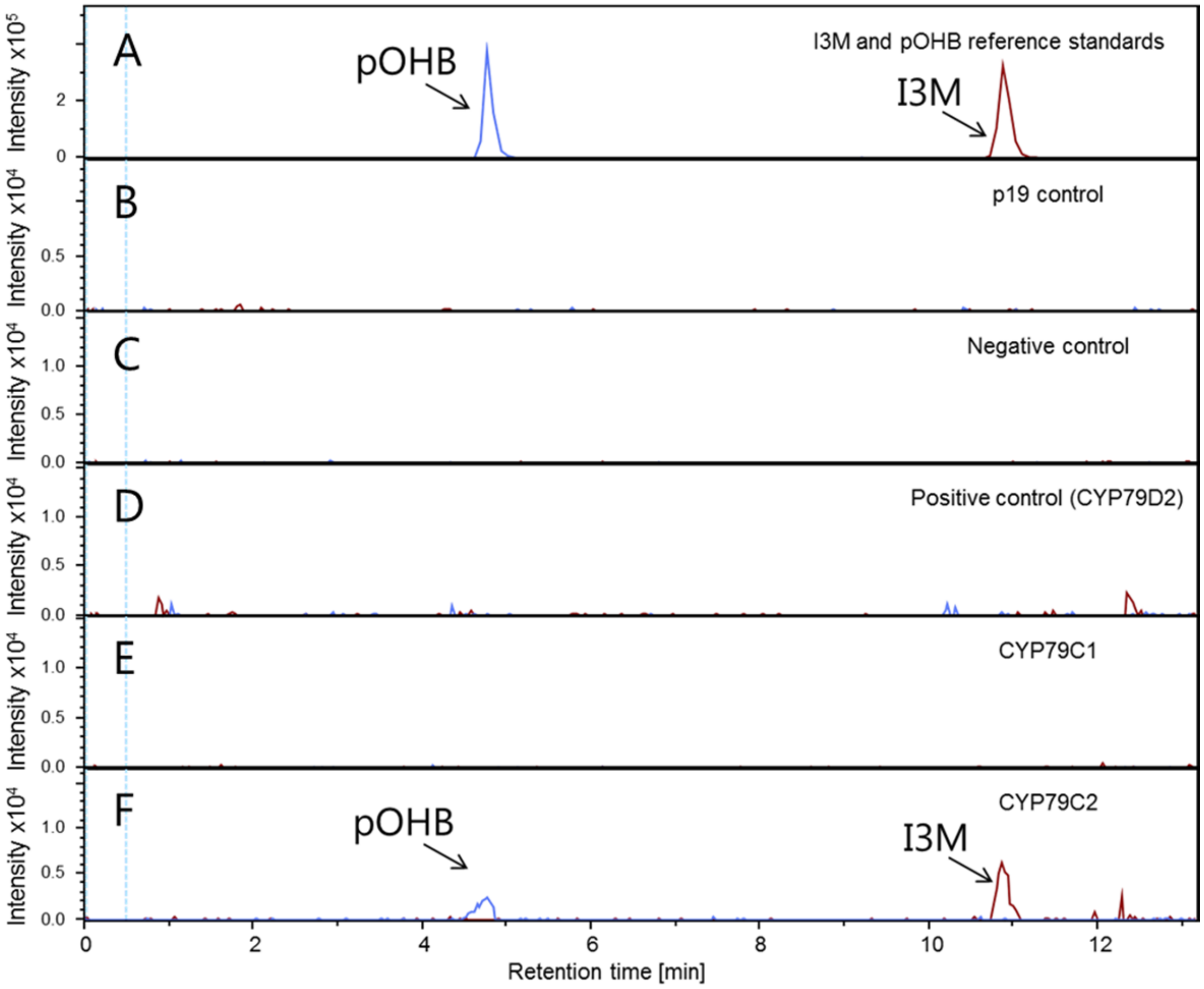

Supplementary Figure S5. Extracted Ion Chromatograms (EIC) of valine-, leucine- and isoleucine-derived intact GLSs (1ME, 2MP and 1MP, respectively) in extracts from *N. benthamiana* after co-expression of CYP79s with the aromatic core pathway. **(A)** Reference standard for 1ME. **(B)** p19 infiltration control. **(C)** Negative control of the aromatic core pathway without any CYP79 co-expression. Presence of 1ME could not be confirmed. **(D)** Positive control of CYP79A2 co-expressed with the aromatic core pathway. **(E)** CYP79C1 co-expressed with the aromatic core pathway and **(F)** CYP79C2 co-expressed with the aromatic core pathway.

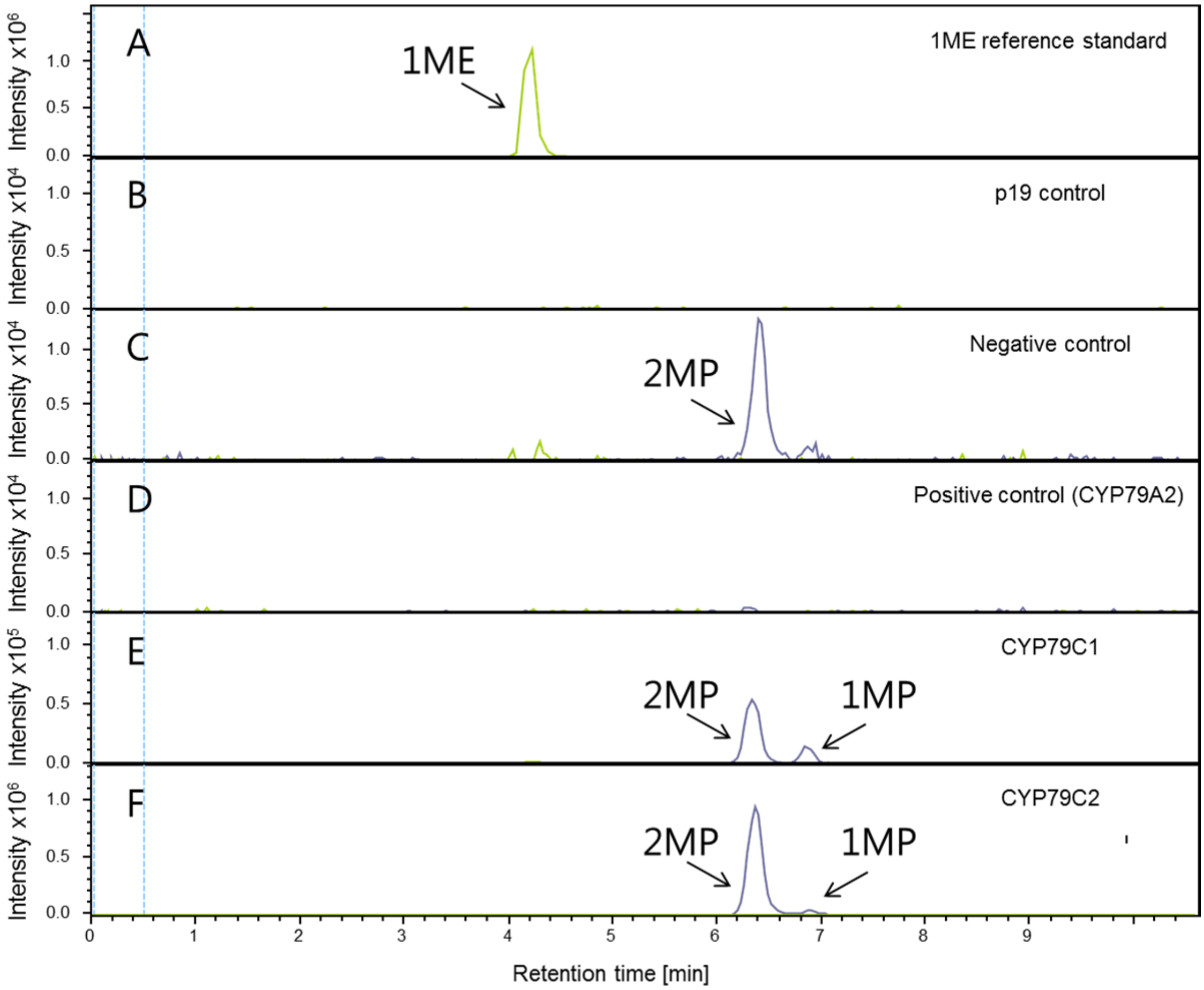

Supplementary Figure S6. Extracted Ion Chromatograms (EIC) of phenylalanine-derived intact GLS (BGLS) in extracts from *N. benthamiana* after co-expression of CYP79s with the aromatic core pathway. **(A)** Reference standard for BGLS. **(B)** p19 infiltration control. **(C)** Negative control of the aromatic core pathway without any CYP79 co-expression. **(D)** Positive control of CYP79A2 co-expressed with the aromatic core pathway. **(E)** CYP79C1 co-expressed with the aromatic core pathway and **(F)** CYP79C2 co-expressed with the aromatic core pathway.

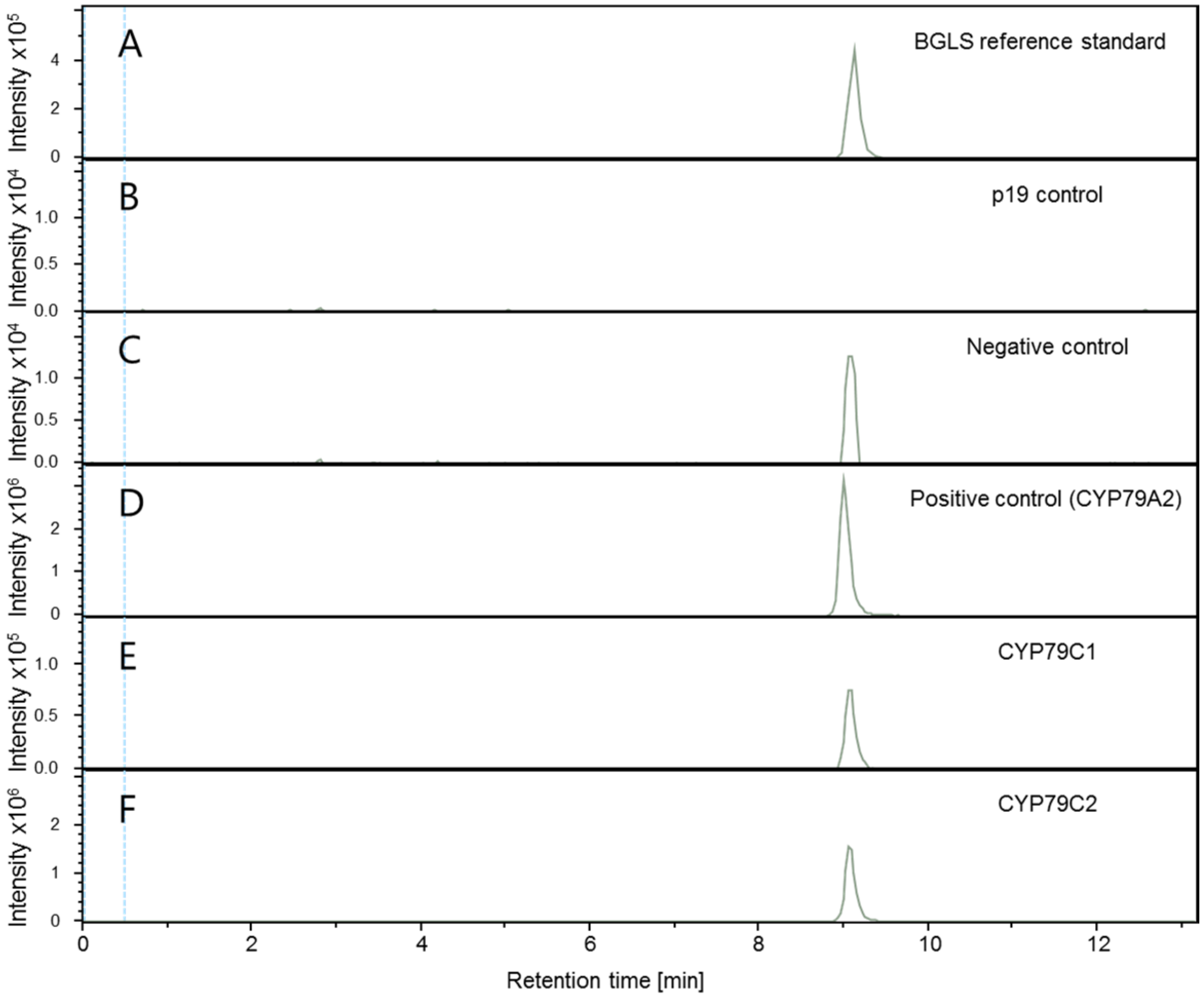

Supplementary Figure S7. Extracted Ion Chromatograms (EIC) of tyrosine and tryptophan-derived intact GLS (pOHB and I3M, respectively) in extracts from *N. benthamiana* after co-expression of CYP79s with the aromatic core pathway. **(A)** Reference standard for pOHB and I3M. **(B)** p19 infiltration control. **(C)** Negative control of the aromatic core pathway without any CYP79 co-expression. **(D)** Positive control of CYP79A2 co-expressed with the aromatic core pathway. **(E)** CYP79C1 co-expressed with the aromatic core pathway and **(F)** CYP79C2 co-expressed with the aromatic core pathway.

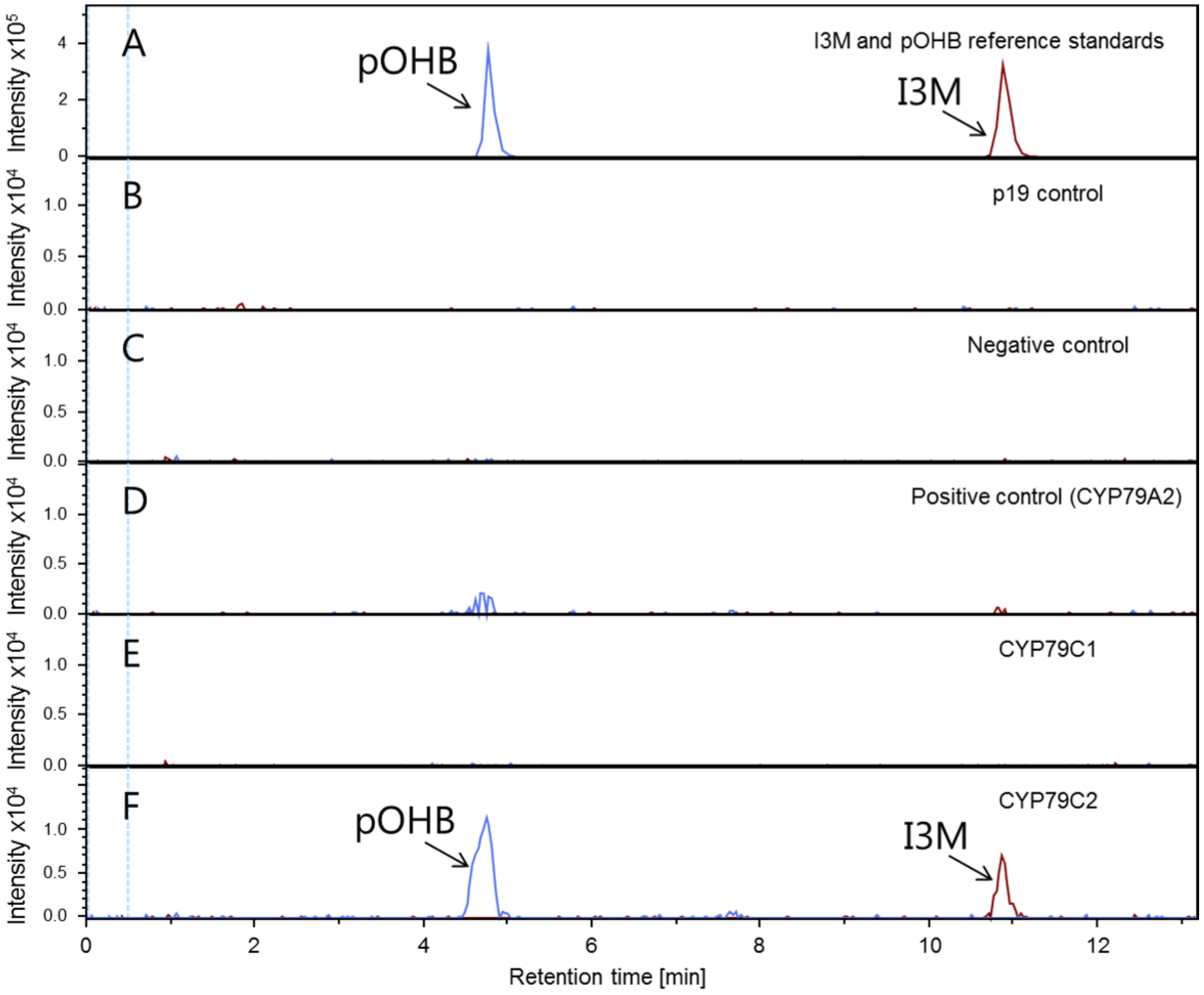

Supplementary Figure S8. LC-MS/QqQ chromatograms traces of valine-derived desulfo-GLS (1ME) in extracts from *N. benthamiana* after co-expression of CYP79s with the aliphatic core pathway. Shown is the transition of ds-1ME GLS from precursor ion to fragment ion (282.1→ 120.0). In contrast to intact GLS analysis by LC-MS/Q-TOF no peak broadening was observed for 1ME from analysis of desulfo-1ME GLS indicating that only 1ME is produced. **(A)** Reference standard for 1ME. **(B)** Positive control of CYP79D2 co-expressed with the aliphatic core pathway. **(C)** CYP79C1 co-expressed with the aliphatic core pathway.

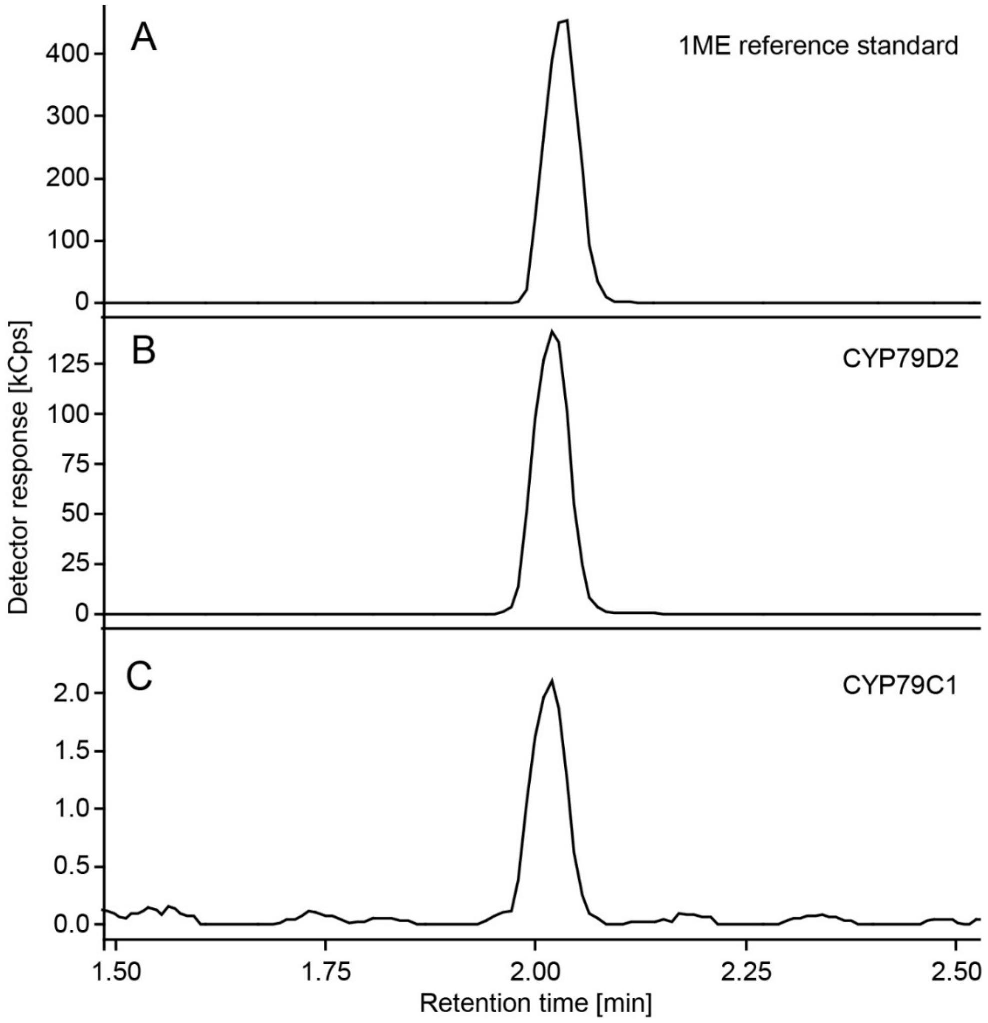

Supplement: Supplementary file 1 [file DataSheet_1.pdf]
