# Supplementary material for: Characterization of Arabidopsis CYP79C1 and CYP79C2 by Glucosinolate Pathway Engineering in Nicotiana benthamiana Shows Substrate Specificity Toward a Range of Aliphatic and Aromatic Amino Acids
Source: Front Plant Sci. 2020 Feb 14;11:57. doi: 10.3389/fpls.2020.00057 (PMC7033466; doi:10.3389/fpls.2020.00057)
Supplement: Supplementary file 2 [file DataSheet_2.pdf]

**Supplementary tables for:**

**Title:** Characterization of Arabidopsis CYP79C1 and CYP79C2 by Glucosinolate Pathway Engineering in *Nicotiana benthamiana* Shows Substrate Specificity Toward a Range of Aliphatic and Aromatic Amino Acids

**Authors:** Cuiwei Wang, Mads Møller Dissing, Niels Agerbirk, Christoph Crocoll and Barbara Ann Halkier1

**Journal**: Frontiers in Plant Science

**Contents:**

**Supplementary Table S1.** Primers in this study.

**Supplementary Table S2:** MRM transitions and response factors for quantification of dsGLSs.

**Supplementary Table S3.** The yield of GLSs produced with the aliphatic core pathway.

**Supplementary Table S4.** *P*-values (Student's *t*-test) used to compare the same type GLS yields between co-expression of *CYP79C1*, *CYP79C2* and no *CYP79* with the aliphatic core pathway.

**Supplementary Table S5.** The yield of GLSs produced with the aromatic core pathway.

**Supplementary Table S6.** *P*-values (Student's *t*-test) used to compare the same type GLS yields between co-expression of *CYP79C1*, *CYP79C2* and no *CYP79* with the aromatic core pathway.

**Supplementary Table S7.** The yield of GLSs produced with the chain elongation pathway and the aliphatic core pathway.

**Supplementary Table S8.** *P*-values (Student's *t*-test) used to compare the GLS yields between co-expression of *CYP79C1* and *CYP79C2* with the chain elongation pathway and the aliphatic core pathway.

**Supplementary Table S1.** Primers in this study.

| Primers | Sequences 5'-3' |
|---|---|
| CW172-CYP79C1-F | GGCTTAAUATGGATTATTATCTGAATAATGTTATCTTCTC |
| CW173-CYP79C1-R | GGTTTAAUTCAAGCTGGACCGGTCCAGAG |
| CW174-CYP79C2-F | GGCTTAAUATGACTATTATCACGAATCCATCC |
| CW175-CYP79C2-R | GGTTTAAUCTAGATCTGGATTTTCGGATAT |
| CW176-Seq-F | GAGTCGACCTGCAGGCAT |
| CW177-Seq-R | GTTTTCCCAGTCACGACGTT |
| CW404-AtCYP79F1 | GGCTTAAUATGATGAGCTTTACCACATCA |
| CW405-AtCYP79F1 | GGTTTAAUTTAAGGACGGAACTTTGGAT |
| CW368-CYP83A1-F | GGCTTAAUATGGAAGATATCATCATCGGCGT |
| CW369-CYP83A1-R | GGTTTAAUCTAATACTTGTTCACTTTCTCTGG |
| CW370-CYP83A1-seq-R | CTTAGGATCAAGCGTCTCATT |
| CW365 -CYP83B1-F | GGCTTAAUATGGATCTCTTATTGATTATAGC |
| CW366 -CYP83B1-R | GGTTTAAUTCAGATGTGTTTCGTTGGT |
| CW367-CYP83B1-seq-R | TTAGGGTCAAGAGTCTCGT |
| CW373-CYP79D2-F | GGCTTAAUATGGCCATGAACGTCTCCA |
| CW374-CYP79D2-R | GGTTTAAUTTAATTCAAGGTGAAGTGGGGT |
| CW376-CYP79D2-seq-R | CGTAATGCCTTGCCGCAAT |
| CW371-GGP1-F | GGCTTAAUATGGTGGAGCAAAAGAGA |
| CW372-GGP1-R | GGTTTAAUCTAGTTAGTTGGAACTCTGCCT |

The primers containing uracil (U) were used for USER cloning and the primers labelled '*seq*' were used for sequencing.

**Supplementary Table S2:** MRM transitions for quantification of selected dsGLSs.

| Analyte $[M+H]^+$ | Retention time [min] | Q1 [*m/z*] | Q3 [*m/z*] | CE [eV] |
|---|---|---|---|---|
| ds1ME GLS | 2.02 | 282.1 | 120.0$^{Qt}$ | 6 |
| | | 282.1 | 86.2 | 19 |
| ds2MP GLS | 2.36 | 296.1 | 134.1 $^{Qt}$ | 7 |
| | | 296.1 | 100.1 | 19 |
| ds1MP GLS | 2.50 | 296.1 | 134.1 $^{Qt}$ | 7 |
| | | 296.1 | 100.1 | 19 |
| dsBGLS | 1.94 | 330.0 | 168.0 $^{Qt}$ | 6 |
| | | 330.0 | 91.0 | 23 |
| ds3MB GLS | 2.94 | 310.0 | 148.0 $^{Qt}$ | 7 |
| | | 310.0 | 114.0 | 19 |
| ds4MP GLS | 3.46 | 324.0 | 162.0 $^{Qt}$ | 7 |
| | | 324.0 | 128.0 | 19 |
| ds allyl GLS | 1.55 | 280.0 | 118.0 $^{Qt}$ | 5 |
| (Internal standard) | | 280.0 | 145.0 | 10 |
| | | 280.0 | 74.2 | 8 |

CE = collision energy; Q = quadrupole; Qt = product ion used for quantification.

**Supplementary Table S3.** The yield of GLSs produced with the aliphatic core pathway.

| | Yield of GLSs (nmol/g fw) | | | | | |
|---|---|---|---|---|---|---|
| | **1ME** | **2MP** | **1MP** | **BGLS** | **pOHB** | **I3M** |
| CYP79C2 | n.d. | 34.67 | 1.00 | 61.40 | n.d. | 0.21 |
| | n.d. | 56.48 | 2.44 | 105.65 | n.d. | 0.27 |
| | n.d. | 28.59 | 1.09 | 61.20 | n.d. | 0.09 |
| | n.d. | 4.49 | 0.21 | 29.30 | n.d. | n.d. |
| | n.d. | 42.73 | 1.72 | 84.02 | n.d. | n.d. |
| | n.d. | 56.30 | 1.70 | 78.23 | n.d. | 0.13 |
| | n.d. | 6.50 | 0.25 | 31.05 | n.d. | 0.13 |
| | n.d. | 31.42 | 0.96 | 100.71 | n.d. | n.d. |
| | n.d. | 51.16 | 1.54 | 134.13 | n.d. | 0.15 |
| Mean±stddev | - | 34.71±19.47 | 1.21±0.72 | 76.19±34.59 | - | 0.16±0.07 |
| CYP79C1 | 0.16 | 0.36 | 0.17 | 2.20 | n.d. | n.d. |
| | 0.12 | 0.59 | 0.15 | 2.74 | n.d. | n.d. |
| | 0.59 | 0.40 | 0.33 | 2.33 | n.d. | n.d. |
| | n.d. | n.d. | n.d. | 0.52 | n.d. | n.d. |
| | 0.11 | 0.30 | 0.10 | 1.36 | n.d. | n.d. |
| | 0.20 | 0.21 | 0.16 | 1.23 | n.d. | n.d. |
| | 0.11 | 0.12 | 0.13 | 1.03 | n.d. | n.d. |
| | n.d. | 0.93 | n.d. | 1.81 | n.d. | n.d. |
| | n.d. | 1.64 | 0.31 | 4.41 | n.d. | n.d. |
| Mean±stddev | 0.14±0.19 | 0.57±0.50 | 0.15±0.11 | 1.96±1.15 | - | - |
| CYP79D2 | 36.05 | 0.24 | 16.90 | n.d. | n.d. | n.d. |
| | 68.93 | 1.47 | 39.40 | n.d. | n.d. | n.d. |
| | 72.93 | 0.62 | 29.70 | n.d. | n.d. | n.d. |
| | 51.83 | 1.04 | 30.14 | n.d. | n.d. | n.d. |
| | 17.78 | 0.38 | 11.59 | n.d. | n.d. | n.d. |
| | 54.31 | 0.77 | 31.96 | n.d. | n.d. | n.d. |
| | 31.47 | 0.47 | 19.54 | n.d. | n.d. | n.d. |
| | 64.54 | 1.59 | 38.91 | n.d. | n.d. | n.d. |
| | 87.00 | 1.26 | 36.40 | n.d. | n.d. | n.d. |
| Mean±stddev | 53.87±22.16 | 0.87±0.49 | 28.28±10.04 | - | - | - |
| No CYP79 | n.d. | n.d. | n.d. | n.d. | n.d. | n.d. |
| | n.d. | n.d. | n.d. | 0.90 | n.d. | n.d. |
| | n.d. | n.d. | n.d. | 0.69 | n.d. | n.d. |
| | n.d. | n.d. | n.d. | 0.35 | n.d. | n.d. |
| | n.d. | n.d. | n.d. | 0.48 | n.d. | n.d. |
| | n.d. | n.d. | n.d. | 0.30 | n.d. | n.d. |
| | n.d. | n.d. | n.d. | 1.25 | n.d. | n.d. |
| | n.d. | n.d. | n.d. | 0.83 | n.d. | n.d. |
| | n.d. | n.d. | n.d. | 1.25 | n.d. | n.d. |
| Mean±stddev | - | - | - | 0.76±0.37 | - | - |

n.d. = not detected, In some cases the detection of very low levels of dsGLS by LC-MS was close to the detection limit. However, the presence of all presented dsGLSs was confirmed by repeated analysis by LC-MS.

**Supplementary Table S5.** The yield of GLSs produced with the aromatic core pathway.

| | **Yield of GLSs (nmol/g fw)** | | | | | |
|---|---|---|---|---|---|---|
| | **1ME** | **2MP** | **1MP** | **BGLS** | **pOHB** | **I3M** |
| CYP79C2 | n.d. | 93.62 | 1.77 | 136.87 | 0.62 | 0.13 |
| | n.d. | 175.87 | 3.83 | 183.49 | 1.17 | 0.22 |
| | n.d. | 101.93 | 2.06 | 191.37 | 0.77 | 0.20 |
| | n.d. | 79.34 | 2.33 | 83.46 | 0.64 | 0.14 |
| | n.d. | 246.58 | 5.15 | 250.77 | 1.72 | 0.31 |
| | n.d. | 328.72 | n.d. | 179.17 | n.d. | 0.40 |
| | n.d. | 145.24 | 2.32 | 150.16 | 0.84 | 0.22 |
| | n.d. | 295.02 | 6.52 | 206.90 | 1.03 | 0.33 |
| | n.d. | 154.91 | 3.96 | 139.21 | 0.95 | 0.19 |
| Mean±stddev | - | 180.14±90.36 | 3.49±1.69 | 169.04±48.13 | 0.97±0.36 | 0.24±0.09 |
| CYP79C1 | n.d. | 4.11 | 0.82 | 4.90 | n.d. | n.d. |
| | n.d. | 14.93 | 1.47 | 3.01 | n.d. | n.d. |
| | n.d. | 6.99 | 1.17 | 2.50 | n.d. | n.d. |
| | n.d. | 2.38 | 0.48 | 2.45 | n.d. | n.d. |
| | n.d. | 10.08 | 1.33 | 2.95 | n.d. | n.d. |
| | n.d. | 17.38 | 1.51 | 4.84 | n.d. | n.d. |
| | n.d. | 2.33 | 0.28 | 2.79 | n.d. | n.d. |
| | n.d. | 3.83 | 0.84 | 3.11 | n.d. | n.d. |
| | n.d. | 5.64 | 1.07 | 2.80 | n.d. | n.d. |
| Mean±stddev | - | 7.52±5.49 | 1.00±0.43 | 3.26±0.94 | - | - |
| CYP79A2 | n.d. | n.d. | n.d. | 231.35 | 0.18 | n.d. |
| | n.d. | n.d. | n.d. | 651.49 | 0.66 | n.d. |
| | n.d. | n.d. | n.d. | 321.57 | 1.11 | n.d. |
| | n.d. | n.d. | n.d. | 346.06 | 0.31 | n.d. |
| | n.d. | n.d. | n.d. | 393.63 | 0.57 | n.d. |
| | n.d. | n.d. | n.d. | 727.56 | 1.47 | n.d. |
| | n.d. | n.d. | n.d. | 256.81 | 0.32 | n.d. |
| | n.d. | n.d. | n.d. | 615.04 | 1.40 | n.d. |
| | n.d. | n.d. | n.d. | 773.88 | 0.87 | n.d. |
| Mean±stddev | - | - | - | 479.71±211.37 | 0.77±0.48 | - |
| No CYP79 | n.d. | 0.86 | n.d. | 1.31 | n.d. | n.d. |
| | n.d. | 0.52 | n.d. | 0.95 | n.d. | n.d. |
| | n.d. | 0.29 | n.d. | 0.93 | n.d. | n.d. |
| | n.d. | 0.20 | n.d. | 0.79 | n.d. | n.d. |
| | n.d. | 0.81 | n.d. | 1.34 | n.d. | n.d. |
| | n.d. | 0.74 | n.d. | 0.90 | n.d. | n.d. |
| | n.d. | 1.04 | n.d. | 2.08 | n.d. | n.d. |
| | n.d. | 0.83 | n.d. | 1.53 | n.d. | n.d. |
| | n.d. | 1.08 | n.d. | 1.25 | n.d. | n.d. |
| Mean±stddev | - | 0.71±0.31 | - | 1.23±0.40 | - | - |

n.d. = not detected In some cases the detection of very low levels of dsGLS by LC-MS was close to the detection limit. However, the presence of all presented dsGLSs was confirmed by repeated analysis by LC-MS.

**Supplementary Table S7.** The yield of GLSs produced with the chain elongation pathway and the aliphatic core pathway.

| | Yield of chain-elongated GLSs (nmol/g fw) | | | | | | |
|---|---|---|---|---|---|---|---|
| | **2MP** | **1MP** | **3MB** | **4MP** | **3MTP** | **4MTB** | **4MSB** |
| CYP79C2 | 1.13 | 0.00 | n.d. | n.d. | n.d. | n.d. | n.d. |
| | 0.88 | 0.00 | n.d. | n.d. | n.d. | n.d. | n.d. |
| | 7.30 | 0.53 | 1.83 | n.d. | n.d. | n.d. | n.d. |
| | 4.00 | 0.38 | 1.94 | 0.45 | n.d. | n.d. | n.d. |
| | 10.10 | 0.70 | 3.75 | 1.25 | n.d. | 0.38 | n.d. |
| | 3.35 | 0.00 | 1.69 | n.d. | n.d. | n.d. | n.d. |
| | 3.82 | 0.00 | 1.43 | 0.53 | n.d. | n.d. | n.d. |
| | 1.92 | 0.00 | 1.04 | n.d. | n.d. | n.d. | n.d. |
| | 8.06 | 0.52 | 2.71 | n.d. | n.d. | n.d. | n.d. |
| | 4.00 | 0.00 | 2.42 | n.d. | n.d. | n.d. | n.d. |
| | 10.06 | 0.46 | 3.76 | 0.96 | n.d. | n.d. | 0.87 |
| | 13.48 | 1.22 | 4.45 | 2.22 | n.d. | 0.79 | n.d. |
| Mean±stddev | 5.68±4.05 | 0.32±0.39 | 2.08±1.42 | 0.45±0.70 | - | 0.10±0.24 | 0.07±0.25 |
| CYP79C1 | n.d. | n.d. | n.d. | 1.11 | n.d. | 3.26 | n.d. |
| | n.d. | n.d. | n.d. | 1.67 | n.d. | 1.60 | n.d. |
| | n.d. | n.d. | n.d. | 2.35 | n.d. | 6.43 | n.d. |
| | n.d. | n.d. | n.d. | 2.06 | n.d. | 5.86 | n.d. |
| | n.d. | n.d. | n.d. | 1.86 | n.d. | 4.13 | n.d. |
| | n.d. | n.d. | n.d. | 1.56 | n.d. | 1.95 | n.d. |
| | n.d. | n.d. | n.d. | 3.55 | n.d. | 2.82 | n.d. |
| | n.d. | n.d. | n.d. | 2.40 | n.d. | 5.14 | n.d. |
| | n.d. | n.d. | n.d. | 1.08 | n.d. | 3.91 | n.d. |
| | n.d. | n.d. | n.d. | 1.13 | n.d. | 2.51 | n.d. |
| | n.d. | n.d. | n.d. | 1.41 | n.d. | 4.63 | n.d. |
| | n.d. | n.d. | n.d. | 0.96 | n.d. | 1.66 | n.d. |
| Mean±stddev | - | - | - | 1.76±0.75 | - | 3.66±1.63 | - |
| CYP79F1 | n.d. | n.d. | 33.26 | 74.66 | 11.02 | 42.87 | 2.08 |
| | n.d. | n.d. | 31.27 | 56.69 | 8.83 | 23.71 | 1.31 |
| | n.d. | n.d. | 73.74 | 129.75 | 7.50 | 21.87 | 1.36 |
| | n.d. | n.d. | 55.78 | 101.79 | 7.50 | 32.63 | 1.69 |
| | n.d. | n.d. | 69.63 | 138.47 | 13.22 | 52.21 | 2.33 |
| | n.d. | n.d. | 61.02 | 122.19 | 11.10 | 38.34 | 2.04 |
| | n.d. | n.d. | 48.16 | 89.70 | 11.59 | 49.78 | 2.27 |
| | n.d. | n.d. | 48.57 | 113.56 | 8.37 | 29.54 | 1.65 |
| | n.d. | n.d. | 64.40 | 97.47 | 9.64 | 28.72 | 1.67 |
| | n.d. | n.d. | 26.63 | 45.79 | 6.57 | 16.22 | 0.87 |
| | n.d. | n.d. | 50.87 | 109.35 | 4.10 | 10.62 | 0.94 |
| | n.d. | n.d. | 47.55 | 116.65 | 15.27 | 49.97 | 2.50 |
| Mean±stddev | - | - | 50.90±15.05 | 99.67±28.57 | 9.56±3.07 | 33.04±13.78 | 1.73±0.53 |
| Negative control | n.d. | n.d. | n.d. | n.d. | n.d. | n.d. | n.d. |
| Only chain elongation pathway | n.d. | n.d. | n.d. | n.d. | n.d. | n.d. | n.d. |
| | n.d. | n.d. | n.d. | n.d. | n.d. | n.d. | n.d. |
| | n.d. | n.d. | n.d. | n.d. | n.d. | n.d. | n.d. |
| | n.d. | n.d. | n.d. | n.d. | n.d. | n.d. | n.d. |
| | n.d. | n.d. | n.d. | n.d. | n.d. | n.d. | n.d. |
| | n.d. | n.d. | n.d. | n.d. | n.d. | n.d. | n.d. |
| | n.d. | n.d. | n.d. | n.d. | n.d. | n.d. | n.d. |
| | n.d. | n.d. | n.d. | n.d. | n.d. | n.d. | n.d. |
| | n.d. | n.d. | n.d. | n.d. | n.d. | n.d. | n.d. |
| | n.d. | n.d. | n.d. | n.d. | n.d. | n.d. | n.d. |
| | n.d. | n.d. | n.d. | n.d. | n.d. | n.d. | n.d. |
| Mean ±stddev | - | - | - | - | - | - | - |

n.d. = not detected. No other GLSs were detected, including BGLS, pOHB and I3M, In some cases the detection of very low levels of dsGLS by LC-MS was close to the detection limit. However, the presence of all presented dsGLSs was confirmed by repeated analysis by LC-MS.

**Supplementary Table S4.** *P*-values (Student's *t*-test) used to compare the same type GLS yields between co-expression of *CYP79C1*, *CYP79C2* and no *CYP79* with the aliphatic core pathway.

| | *P*-values (Student's *t*-test) for comparison of GLS yields by expression of aliphatic core structure pathway | | | | | | | | | |
|---|---|---|---|---|---|---|---|---|---|---|
| | 1ME | | 2MP | | 1MP | | BGLS | | I3M | |
| | CYP79C2 | CYP79C1 | CYP79C2 | CYP79C1 | CYP79C2 | CYP79C1 | CYP79C2 | CYP79C1 | CYP79C2 | CYP79C1 |
| No CYP79 | - | 0.05 | < 0.001 | 0.01 | < 0.001 | < 0.001 | < 0.001 | 0.01 | 0.01 | - |

**Supplementary Table S6.** *P*-values (Student's *t*-test) used to compare the same type GLS yields between co-expression of *CYP79C1*, *CYP79C2* and no *CYP79* with the aromatic core pathway.

| | *P*-values (Student's *t*-test) for comparison of GLS yields by expression of aromatic core structure pathway | | | | | | | | | |
|---|---|---|---|---|---|---|---|---|---|---|
| | 2MP | | 1MP | | BGLS | | pOHB | | I3M | |
| | CYP79C2 | CYP79C1 | CYP79C2 | CYP79C1 | CYP79C2 | CYP79C1 | CYP79C2 | CYP79C1 | CYP79C2 | CYP79C1 |
| No CYP79 | < 0.001 | 0.01 | < 0.001 | < 0.001 | < 0.001 | < 0.001 | < 0.001 | - | < 0.001 | - |

**Supplementary Table S8.** *P*-values (Student's *t*-test) used to compare the GLS yields between co-expression of *CYP79C1* and *CYP79C2* with the chain elongation pathway and the aliphatic core pathway.

| | *P*-values (Student's *t*-test) for comparison of GLS yields between expression of *CYP79C1* or *CYP79C2* with the chain elongation and aliphatic core pathways | | | | | | |
|---|---|---|---|---|---|---|---|
| | 2MP | 1MP | 3MB | 4MP | 3MTP | 4MTB | 4MSB |
| *P*-values | < 0.001 | 0.02 | < 0.001 | < 0.001 | - | < 0.001 | 0.34 |
